# College and the “Culture War”: Assessing Higher Education’s Influence on Moral Attitudes

**DOI:** 10.1177/00031224211041094

**Published:** 2021-09-18

**Authors:** Miloš Broćić, Andrew Miles

**Affiliations:** a University of Toronto

**Keywords:** moral attitudes, higher education, culture war, socialization, political sociology

## Abstract

Moral differences contribute to social and political conflicts. Against this backdrop, colleges and universities have been criticized for promoting liberal moral attitudes. However, direct evidence for these claims is sparse, and suggestive evidence from studies of political attitudes is inconclusive. Using four waves of data from the National Study of Youth and Religion, we examine the effects of higher education on attitudes related to three dimensions of morality that have been identified as central to conflict: moral relativism, concern for others, and concern for social order. Our results indicate that higher education liberalizes moral concerns for most students, but it also departs from the standard liberal profile by promoting moral absolutism rather than relativism. These effects are strongest for individuals majoring in the humanities, arts, or social sciences, and for students pursuing graduate studies. We conclude with a discussion of the implications of our results for work on political conflict and moral socialization.

Between 2015 and 2019, the number of Republicans who believe higher education has a negative effect on the United States increased by 22 percent (Parker 2019). This uptick is recent, but conservative apprehension toward higher education is not. Conservative critiques vary in the details, but all claim that higher education is promoting progressive worldviews at the expense of conservative orthodoxy. As early as 1959, conservative pioneer Russell Kirk (1959:428) argued that college students are being “defrauded by social indoctrination in the guise of scholarship.” Years later, Allan Bloom attacked universities for spreading a corrosive moral relativism in his best-selling *The Closing of the American Mind* (1987). Claims such as these have motivated a substantial body of research aimed at assessing the extent to which colleges and universities shape political attitudes and identities and have fueled larger concerns over cultural conflict in the United States (Campbell and Horowitz 2016; Elchardus and Spruyt 2009; Hunter 1991; Newcomb 1943; Strother et al. 2020).

Although nominally political, many scholars argue that conflicts such as those surrounding higher education are rooted in differing moral visions (Haidt 2012; Hunter 1991; Miles and Vaisey 2015). Conservatives and liberals display different profiles of moral concerns, with liberals placing a greater emphasis on moral relativism and concern for others, and conservatives emphasizing fixed moral standards, social order, and personal purity (Graham, Haidt, and Nosek 2009; Haidt 2012; Hunter 1991; Koleva et al. 2012; Miles and Vaisey 2015). Higher education’s influence on these moral concerns, however, is unclear. Some contemporary accounts depict universities as puritanically committed to a liberal “culture of victimhood” (Campbell and Manning 2018; Lukianoff and Haidt 2018; Pluckrose and Lindsay 2020), a fact seemingly at odds with previous critiques of “permissive” moral relativism (Hunter 1991; Wuthnow 1989). Recent scholarship also questions whether higher education has a meaningful causal effect on attitudes at all, stressing the role of selection processes instead (Campbell and Horowitz 2016; Gross 2013; Mayrl and Uecker 2011). These difficulties are compounded by claims that moral worldviews crystallize early in life and remain mostly settled (Kiley and Vaisey 2020; Vaisey and Lizardo 2016). Despite being a visible arena for cultural conflicts, higher education’s influence on moral attitudes remains unclear.

The current study addresses this gap by assessing how higher education influences moral attitudes using four waves of the National Study of Youth and Religion, a large, nationally representative dataset that follows respondents from high school into young adulthood. These data cover the period during which most respondents pursue higher education, making them well-suited to answering our question. We begin by briefly reviewing moral conflict in the United States and its relation to higher education. We then discuss higher education’s effect on attitudes, and the challenges that *moral* attitudes pose to this narrative. Finally, we assess the role of higher education in shaping moral attitudes. We find that higher education often shifts moral concerns in a liberal direction, but that for most students these changes are accompanied by a rise in moral certainty rather than relativism. We conclude by discussing how our findings relate to changes in higher education, and the implications for partisan political conflict and moral socialization.

## Moral Conflict in the United States

When discussing moral conflict in the United States, scholars often use a “culture war” framing—a term frequently associated with the rise of the New Christian Right during the 1980s and that gained traction in conservative media. The dramatic nature of the term, however, disguises a more nuanced reality. Gross, Medvetz, and Russell (2011) suggest the culture war takes place primarily in the “civil sphere” (Alexander 2006)—its importance is symbolic, shaping the discourse that political and media actors draw on, but it is not an accurate portrayal of public opinion. Consistent with this, evidence suggests polarization on political issues is largely confined to elites, and most people are relatively moderate in their views and support both conservative and liberal positions (Baldassarri and Gelman 2008; DiMaggio, Evans, and Bryson 1996; Fiorina 2017; Hunter and Wolfe 2007). Yet even without extreme public polarization, meaningful political differences exist that affect how people vote, who they are friends with, and even how they feel about those on the opposite side of the political spectrum (Iyengar and Krupenkin 2018; Mason 2018; for a review, see Iyengar et al. 2019). More importantly, efforts to prove or disprove the existence of a culture “war” often distract from a fundamental and socially significant claim of culture war theories: that moral differences are at the heart of many social and political conflicts.

Scholars have proposed several theories describing how morality leads to differing political attitudes, identities, and behaviors. Hunter (1991) claims conflict stems from competing moral epistemologies. Orthodoxy sees moral truth as coming from an “external, definable, and transcendent authority” that provides fixed standards for behavior (Hunter 1991:44). Progressivism, on the other hand, regards moral truth as relative and subject to revision according to the evolving needs of humans and societies. Orthodoxy is generally associated with political conservatism, and progressivism is tied to liberalism. While these differences largely pertain to the *form* of beliefs—particularly the transcendent versus contextual basis of morality—more recent accounts focus on differences in the *content* of moral concerns. Moral Foundations Theory (MFT) posits five innate psychological foundations that trigger automatic gut-reactions and motivate judgments of right and wrong (Haidt 2012). These include the “individualizing” foundations of care and justice—individualizing in that they privilege the well-being of individuals—and the “binding” foundations of loyalty, authority, and sanctity that serve to uphold social order. Conservatives in the United States endorse individualizing and binding foundations about equally. Liberals emphasize individualizing foundations somewhat more strongly than conservatives and place much less weight on binding foundations (Graham et al. 2009; Koleva et al. 2012).

Miles and Vaisey (2015) compared several theories of morality and politics and found support for both Hunter’s theory and MFT: beliefs about moral relativism and moral concerns focused on the well-being of others and social order explained a third of variation in political ideology. The division between other-focused and order-focused moral concerns was shared by almost all the theories examined. Given this, we propose that concern for others, concern for social order, and views on moral relativism are dimensions of morality that are particularly consequential for explaining morally-motivated social conflict. Accordingly, our analyses focus on moral attitudes related to these dimensions.

## Moral conflict in U.S. Higher education

Higher education has been at the forefront of moral conflict since at least the 1960s (Hunter 1991; Inglehart 2018; Wuthnow 1988). Moral conflict previously occurred mostly within and among religious denominations, but the immense expansion of higher education during the 1960s supplanted these differences and restructured moral conflict along levels of education (Putnam and Campbell 2010; Wuthnow 1988). Egalitarian values concerned with minority group rights became a hallmark of the growing college-educated class, distinguishing them from the “outmoded” or “bigoted” traditionalism of the less educated. Growing recognition of diverse cultures and alternative norms among the college-educated gave cultural preferences a sense of relativity that broke from traditional orthodoxy. Reacting against these changes, conservative churches mobilized to defend traditional ways of life, with the “New Christian Right” leading the backlash against “amoral” liberal positions on issues related to gender and sexuality, as well as the “permissive” effects of moral relativism more generally (Gross et al. 2011; Wuthnow 1988). Without transcendental justification, conservatives feared relativism paved the way for moral laxity and unbridled hedonism.

Developments in higher education during the 1980s elicited criticism of relativism for different reasons. Emerging programs in race, gender studies, and related fields drew on postmodern intellectual currents that questioned the foundations of objective knowledge. Rather than moral permissiveness, cultural critics became increasingly concerned that relativism devolved into dogmatism: by rejecting independent knowledge, relativism reduced truth to a function of group membership, fomenting an unequivocal commitment to identity politics (Bloom 1987; Lasch 1994). Concerns over the educated class’s increasingly *puritanical* rather than *permissive* moralism continued in debates over “political correctness” into the 1990s (D’Souza 1991; Kimball 1990). As moral conflict of this sort became more salient, partisan politics realigned accordingly. Democrats shifted their appeal to the cultural politics of the college-educated, gradually reversing the educational cleavage on voting: the college-educated disproportionately voted Republican before the 1970s, but they came to mostly vote Democrat by the 2000s (Piketty 2020).

Recent accounts indicate that trends toward identity-based morality may have evolved into a “culture of victimhood” on college campuses (Campbell and Manning 2018; Lukianoff and Haidt 2018; Pluckrose and Lindsay 2020). According to Campbell and Manning (2018), victimhood culture grants moral status to those who suffer, valorizes those who vigilantly monitor conduct for signs of oppression, and treats opposition to its ideals as severe offenses. Consequently, some college campuses are reportedly awash in “vindictive protectiveness” that forces students, staff, and faculty alike to “think twice before they speak up, lest they face charges of insensitivity, aggression, or worse” (Lukianoff and Haidt 2015). Systematic evidence for these claims is still sparse, so it is not yet clear how widespread victimhood culture is, nor how accurately these accounts reflect moral attitudes of rank-and-file students. However, these developments raise the intriguing possibility that higher education encourages a modified liberal morality: although the college-educated share a high level of concern for others and relatively low concern for traditional social order, they depart from the common liberal profile by infusing their beliefs with a sense of moral certainty, which is seemingly at odds with an emphasis on moral relativism.

## Higher Education and Moral Change

Strong claims about the influence of higher education have provoked considerable research on whether higher education meaningfully changes students’ attitudes. However, most of this research examines political attitudes rather than moral attitudes directly. What research on morality exists primarily focuses on how higher education shapes moral reasoning (Maxwell and Narvaez 2013; Mayhew et al. 2016). Studies find that higher education encourages individuals to move from basing their moral judgments on personal interest and blind allegiance to social norms toward more critical and universally applied principles of justice (Mayhew et al. 2016; Parker et al. 2016). This focus on universal justice bears a strong resemblance to liberal concerns with social justice, giving credence to the idea that higher education liberalizes morality.

Work on political attitudes gives further insight into how higher education might influence morality. Scholars generally contend that higher education has a liberalizing effect on political attitudes but differ on whether this change is universal (Astin, Astin, and Lindholm 2010; Hanson et al. 2012; Weil 1985). One position is that higher learning increases students’ ability to manage complexity, engage in abstract thinking, and take the perspectives of others (Stubager 2008; Van de Werfhorst and de Graaf 2004). These enhanced cognitive abilities purportedly lead to lower levels of outgroup prejudice and more liberal attitudes generally (Adorno et al. 1950; Altemeyer 1996; Bobo and Licari 1989; Hyman and Wright 1979; Jost et al. 2003). This view is commonly known as the “cognitive hypothesis.” Other scholars endorse a “socialization hypothesis” that argues change is contingent on the transmission of norms, commonly through a particular field of study (Dey 1996; Sidanius et al. 2003; Weil 1985; Zhang and Brym 2019). In this view, fields of study act as subcultures with implicit normative approaches promoted through curricular content (Ladd and Lipset 1975; Parker et al. 2016; Sidanius et al. 2003). Degrees in the liberal arts expose students to cultural diversity in ways that can promote social empathy, curiosity, and critical orientations that in turn foster liberal political attitudes (Parker et al. 2016; Stubager 2008; Van de Werfhorst and de Graaf 2004). Disciplines like business or agriculture, by contrast, focus on solving immediate problems within existing social arrangements (Ladd and Lipset 1975; Stubager 2008; Van de Werfhorst and de Graaf 2004). This could reinforce the legitimacy of existing social orders and might promote conservative attitudes that defend the status quo (Parker et al. 2016; Sidanius et al. 2003).

Applied to morality, the cognitive hypothesis suggests higher education will liberalize moral sensibilities by developing students’ cognitive sophistication. Students’ empathy will increase as they learn to take the role of the other (Kohlberg 1984; Piaget and Gabain 1965), and exposure to moral inconsistencies across cultures and historical periods will provoke skepticism of tradition (Campbell 2017). Students may therefore adopt more relativistic attitudes that divest tradition of moral sanctity in an effort to dismantle perceived barriers to individual justice. The socialization hypothesis, however, suggests moral liberalization is primarily a by-product of learning specific course content, with the liberalizing effect mostly confined to students majoring in the social sciences, humanities, or other liberal arts fields (Ladd and Lipset 1975; Parker et al. 2016; Sidanius et al. 2003).

An alternative version of the socialization hypothesis suggests how universities might promote moral certainty rather than (or in addition to) relativism. In this view, students do not passively grow into moral relativism through exposure to human diversity but are actively taught the virtue of particular beliefs that come to be seen as institutionally sanctioned (Collins 1971; Jackman and Muha 1984; Mills 1956). This has sometimes been understood as “conservatizing” students into the status quo, but recent developments suggest these “official” beliefs are increasingly shifting toward liberal moral concerns, particularly in the humanities, arts, and social sciences. Pluckrose and Lindsay (2020), for instance, argue that activist scholarship in interdisciplinary fields such as postcolonial theory and gender studies often asserts *theoretical* claims amenable to social justice activism as objectively *true* statements about the social world. Smith (2014) advances similar conclusions in his reflection on American sociology, arguing that sociologists are engaged in a “sacred project” aimed at achieving individual emancipation, self-determination, and personal affirmation for all people (cf. Martin 2016). These claims are consistent with a recent survey of 479 U.S. sociologists that found a majority of respondents endorsed the idea that sociology has a moral mission (Horowitz, Haynor, and Kickham 2018). Moral/political motivations have also been linked to resistance to evolutionary explanations in psychology (von Hippel and Buss 2018), the near exclusive focus among social psychologists on conservative (rather than liberal) prejudice (Crawford 2018), and the heavy focus on bias (rather than accuracy) in studies of social perception (Jussim 2012). Collectively, this research suggests that—at a minimum—a substantial minority of scholars in the humanities and social sciences view their work in moral terms. To the extent this is true, it follows that students in these fields will be exposed to liberal moral viewpoints. These views could foster a sense of moral certainty insofar as students view them as based in expertise or scientifically validated facts.

## Challenges to Moral Change

The cognitive and socialization hypotheses both suggest higher education has real effects on political attitudes and—by extension—on moral attitudes. However, any account of moral change motivated by work on political attitudes faces at least two challenges. First, moral attitudes are not equivalent to political attitudes and might be less amenable to change. Research on morality suggests moral concerns are learned early in life and rely on evolutionarily shaped predispositions. Some moral instincts (e.g., a preference for prosociality) seem to be present from birth (Hamlin, Wynn, and Bloom 2007; Hamlin et al. 2011; Killen and Smetana 2015; Warneken 2013; cf. Haidt 2012). Morality is further refined during childhood and adolescence as individuals interact with families and peers and learn to navigate institutional settings (for a review, see Killen and Smetana 2015). The early development of morality might explain Vaisey and Lizardo’s (2016) finding that changes in political attitudes are closely tied to time periods, and moral attitudes are more closely tied to cohorts. Vaisey and Lizardo argue that moral attitudes are deeply internalized during early-life socialization and form lasting differences across cohorts. Political attitudes, by contrast, respond to changes in the prevailing zeitgeist and are more susceptible to period effects. Given that many moral attitudes seem to be tied to innate intuitions and early moral learning, there might be limited room for moral change by the time individuals enroll in higher education.

A second issue is that higher education’s effects are often driven by selection processes. Selection processes have been found to account for higher education’s effects on political engagement (Jennings and Stoker 2008), civic engagement (Schnittker and Behrman 2012), ideological leaning (Campbell and Horowitz 2016; Elchardus and Spruyt 2009), religiosity (Mayrl and Uecker 2011), and earning outcomes (Dale and Krueger 2014; Zhou 2019). Moral change might likewise be an artifact of selection, with people who enroll in higher education already differing in their moral commitments from those who do not. For example, individuals from families with high socioeconomic status (SES) are more likely to pursue higher education (Blau and Duncan 1967; Campbell and Horowitz 2016; Conley 2001; Schnittker and Behrman 2012) and to express principles consistent with a liberal moral profile than are individuals from families with fewer social and economic resources (Lamont et al. 1996; Longest, Hitlin, and Vaisey 2013; Miles 2014; Sayer 2010; Vaisey and Miles 2014).^1^ Individuals might also self-select into pursuing greater higher education based on their moral attitudes (Mitchell et al. 2008; Vaisey 2010). This is especially true following enrollment, where personal values influence decisions about the extent and direction of continuing studies (Mullen, Goyette, and Soares 2003; Stolzenberg 1994). Gross (2013), for instance, argues that liberal students disproportionately choose to pursue advanced training because they perceive an affinity between their values and the liberal academic environment, whereas conservative students opt for other careers to avoid value conflict. In summary, selection processes based on either family background or prior moral attitudes might make any apparent effects of higher education on moral attitudes spurious.

## The Current Study

Research to date provides no clear answers about how higher education changes moral attitudes. Existing work suggests colleges and universities will generally liberalize moral concerns—that is, increase concern for others and reduce concern for social order. What is less clear is whether these liberal views will be accompanied by a growing sense of moral relativism, moral certainty, or some combination of the two. This narrative is further complicated by research highlighting that morality is learned early in life, and by the possibility that individuals select into higher education based on their family background or preexisting moral attitudes. Consequently, it remains unclear *how* higher education influences moral attitudes, and *whether* it does so at all.

We address these questions using four waves of data from the National Study of Youth and Religion (NSYR). The NSYR follows respondents from adolescence to early adulthood and contains measures of educational attainment and field of study, as well as variables capturing moral relativism, moral concern for others, and moral concern for social order. The data also contain a rich array of family background variables that can be used to control for selection into higher education and fields of study.

## Methods

### Data

The NSYR is a four-wave, nationally representative survey study that investigates the beliefs and practices of U.S. youth. The first wave of data was collected from June 2002 to April 2003 and included 3,370 teenagers between the ages of 13 and 17. During this first wave, a parent of each respondent was also interviewed. Waves 2, 3, and 4 were collected in 2005, 2007–2008, and 2012–2013, respectively, with respondents being between 23 and 29 years old during the final wave. Sample sizes for each analysis and our strategies for handling missing data are described below.

### Measurements

#### Moral attitudes

We measure moral relativism with two questions from waves 2, 3, and 4.^2^ The two items are moderately correlated (*r_wave 2_* = .42; *r_wave 3_* = .37; *r_wave 4_* = .44), but the theoretical considerations given above suggest they might respond to higher education differently, so we examine them separately. The first question asks respondents to rate their level of agreement with the following statement: “[m]orals are relative, that there are no definite rights and wrongs for everybody.” This question taps a general belief in *moral relativism*. The second question asks for agreement with the statement, “[t]he world is always changing and we should adjust our views of what is morally right and wrong to reflect those changes.” We refer to this dimension as *moral progressivism*. Responses for both items include strongly disagree, disagree, agree, and strongly agree.

We measure concern for others and for social order using the 20-item short-form of the cross-nationally validated Moral Foundations Questionnaire (MFQ20), which was only included at wave 4 (Doğruyol, Alper, and Yilmaz 2019; Graham et al. 2011). Moral concern for others is operationalized using the average scores of the individualizing foundations of care/harm and fairness/cheating (α = .71). Concern for social order is measured as the average scores of the binding foundations of loyalty/betrayal, authority/subversion, and sanctity/degradation (α = .79). Further coding details can be found in Appendix A, and analyses for individual moral foundations are in Appendix Table D1.

#### Higher education

We measure education in two ways to capture the time spent in higher education and exposure to different programs of study. We measure the total amount of higher education completed using dichotomous indicators for whether or not respondents completed some college, received a bachelor’s degree, or are pursuing a graduate/professional degree, with respondents who only received a high school degree/GED/no degree as the reference category.^3^ Field of study was asked at wave 4 in open-ended fashion of all respondents who pursued postsecondary education. Following past work, we grouped academic disciplines by categorizing text entries for current majors and the major of a respondent’s first bachelor’s degree into five groups: humanities, arts, and social science (HASS, *n* = 494); STEM (*n* = 364); business and agriculture (*n* = 317); and primary or secondary education (*n* = 58). These categories are not mutually exclusive; respondents who listed double majors in different areas were coded as 1 in each. The reference category for these variables is respondents not enrolled in higher education and who consequently were not exposed to a particular field of study (*n* = 459). The few respondents who reported majors that did not fit in any of these categories were omitted from the sample (*n* = 8). Coding details for fields of study can be found in Appendix Table A1.

### Analytic Strategy

Our analysis proceeds in several steps. We begin by describing how our two measures of moral relativism vary across levels of educational attainment and field of study during wave 4, and then proceed to multivariable analyses. Multivariable models include age to control for possible age-related confounders (see Peltzman 2019), and for agreement with the statement, “it is sometimes okay to break moral rules if it works to your advantage and you can get away with it.” The latter control allows us to isolate true moral relativism from justifications for deviance. We include any respondent who completed wave 2, 3, or 4, leaving us with 3,231 respondents. We adjust for missing data using full-information maximum likelihood estimation, use cluster-robust standard errors to adjust for dependencies among observations, and do not include sampling weights to increase the precision of estimates (Enders 2010; Solon, Haider, and Wooldridge 2015).^4^

The fact that moral relativism items are available at waves 2, 3, and 4 makes it possible to use linear fixed-effect estimation to control for all time-invariant characteristics (e.g., family background) whose effects do not vary over time. However, fixed-effects estimation reduces the amount of information used in estimating effects by removing between-person variation (Allison 2009). This reduces statistical power and—when the loss of information is severe—estimates may become unreliable (see Appendix B). Our analyses involve simultaneously estimating the effects of multiple educational attainment categories and fields of study, which could stretch the data even thinner. Consequently, there is a need to safeguard statistical power to maximize the chance of detecting cross-category differences. We thus test whether fixed-effects estimation is necessary. To do so, we use the correlated random-effects model (CRE; Wooldridge 2016), which is statistically equivalent to Allison’s (2009) hybrid random-effects model:

(1) $$y_{it}=\beta_{0}+\beta x_{it}+\gamma {\bar{x}}_{i}+r_{i}+e_{it}.$$

Here the outcome *y* varies over individuals (*i*) and time (*t*). The ***β*** are fixed-effects estimates of the time-varying variables (*x_it_*), and the ***γ*** are the effects of the within-person, cross-time means of those variables. The error term consists of time-varying (*e_it_*) and time-invariant (*r_i_*) influences that are uncorrelated with the time-varying *x_it_*. Time-constant predictors can be included but are not shown in Equation 1. Importantly, the CRE allows the FE assumption to be tested on a variable-by-variable basis by assessing whether each coefficient in ***γ*** is significantly different from 0. FE estimation can then be eliminated for variables where it is not needed and replaced with random-effects estimation. See Appendix B for a fuller discussion of this method.

After examining higher education’s effects on moral relativism, we examine its effects on specific moral content—in particular, on moral concern for others and for social order. The NSYR does not include the MFQ20 at waves 1, 2, and 3, preventing the use of CRE models to control (or test) for time-constant unobserved confounds. We therefore use multivariable linear models to control for selection effects. All control variables were measured at wave 1 and thus predate university enrollment. We included controls based on prior research addressing selection into higher education and field of study. Preliminary models predicting college enrollment, level of educational attainment, or major choice in our sample corroborate the importance of these controls (for full details, see Appendix C). Controls include measures of family socioeconomic background, ethnicity, religious affiliation, parental political ideology, immigrant status, parental expectations for children’s education, respondents’ educational aspirations, high school grades, gender, and variables related to personality. To capture potential self-selection based on preexisting moral concerns, we include measures related to moral concern for others and social order, such as social concern for marginalized populations, attitudes toward premarital sex, and religiosity. To account for non-random sample attrition, we include controls that predict whether respondents participated in wave 4.

Coding details for all control variables can be found in Appendix A. All models are adjusted for missing data using full-information maximum likelihood estimation (Enders 2010). We standardized scores for the moral concern scales and all non-dichotomous predictors prior to analyses so that education effects can be interpreted in standard deviation (SD) units. We exclude all respondents who did not participate at wave 4, leaving a final sample of *N* = 2,012.

Finally, we examine the scope of higher education’s effects by testing whether effects are consistent for students who come from ideologically diverse households, and by using model-based predictions to assess the moral outcomes of different pathways through higher education.

Taken together, our analyses aim to estimate the causal effect of higher education on moral attitudes. Such attempts are always provisional and rely on the assumption that the methodological techniques used adequately account for alternative explanations. Our data allow for stronger inferences about moral relativism and progressivism given our ability to test for unobserved confounding, but our claims about moral concerns for others and social order are comparatively weaker given that these constructs are only measured at wave 4 and must therefore rely more heavily on explicit controls. Our covariates are not exhaustive, but they are sufficiently rich to make causal interpretation plausible. In short, although our analysis cannot conclusively establish causality, our methods make causal interpretation credible, albeit to different degrees. We return to this issue in the Discussion section.

## Results

### Moral Relativism and Higher Education

**Figure 1.**
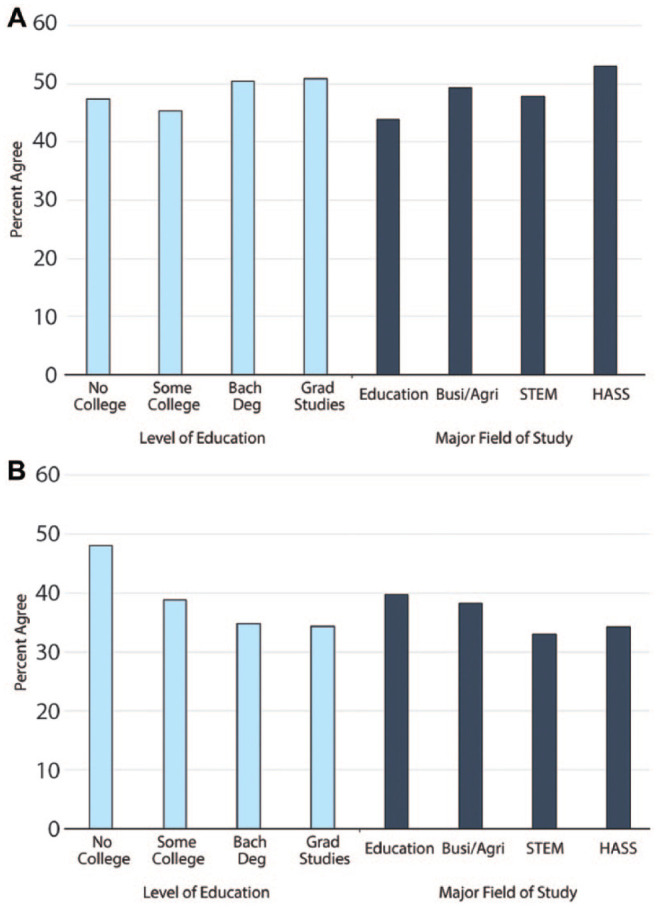
Panel A: Percent Agree (agree/strongly agree) on Moral Progressivism by Educational Attainment and Major Field of Study; Panel B: Percent Agree (agree/strongly agree) on Moral Relativism by Educational Attainment and Major Field of Study

We begin by describing patterns of moral progressivism and moral relativism at wave 4. Figure 1 plots proportions of respondents who either agreed or strongly agreed that morals should change as societies progress (moral progressivism) and that there is no absolute moral truth (moral relativism). Proportions are shown by levels of education and fields of study. Consistent with the cognitive hypothesis, moral progressivism is greater among degree-holders than for others. The socialization hypothesis also receives support insofar as moral progressivism varies by field of study and is most pronounced among graduates of the humanities, arts, and social sciences (HASS).^5^ In contrast—and contrary to early conservative critiques—higher educational attainment is associated with *less* moral relativism, especially for individuals majoring in STEM or HASS fields.

We next turn to multivariable analyses to address the possibility that these descriptive patterns are due to selection effects or the influence of other unobserved, time-constant influences. Table 1 tests whether this is the case by presenting results from CRM models for moral progressivism and moral relativism (Models 1 and 3). In both cases, none of the cross-wave means are significantly different from 0, suggesting that confounding from time-invariant variables is not severe and justifying the use of the more statistically powerful random-effects estimation. In the present case, random-effects estimation may also give less biased estimates (for details, see Appendix B).

**Table 1.** Correlated Random Effects and Random-Effects Linear Models of Moral Relativism and Higher Education

	Moral Progressivism: “World is always changing . . . adjust our views of what is morally right and wrong”				Moral Relativism: “Morals are relative . . . no definite rights and wrongs for everybody”			
	1. CRE		2. RE		3. CRE		4. RE	
Variables	Coef.	SE	Coef.	SE	Coef.	SE	Coef.	SE
Highest Level of Education								
Some College	.051	(.057)	.026	(.031)	−.022	(.063)	−.014	(.030)
Bachelor’s Degree	.072	(.079)	.034	(.056)	−.093	(.084)	−.079	(.055)
Graduate Studies	.205^*^	(.090)	.137^*^	(.070)	−.162	(.096)	−.124	(.070)
Major Field of Study								
HASS	.238^**^	(.088)	.096^*^	(.039)	−.212^*^	(.088)	−.112^**^	(.036)
STEM	.035	(.091)	−.035	(.043)	.063	(.096)	−.118^**^	(.040)
Business/Agriculture	−.003	(.098)	.050	(.045)	.005	(.097)	−.057	(.040)
Education	.238	(.157)	.023	(.095)	−.064	(.214)	−.098	(.084)
*Cross-Wave Effects*								
Highest Level of Education								
Some College	−.056	(.067)			−.009	(.071)		
Bachelor’s Degree	−.131	(.126)			−.001	(.127)		
Graduate Studies	−.217	(.145)			.054	(.146)		
Major Field of Study								
HASS	−.125	(.097)			.120	(.095)		
STEM	−.006	(.104)			−.161	(.104)		
Business/Agriculture	.093	(.108)			−.062	(.108)		
Education	−.204	(.188)			−.039	(.227)		
*Controls*								
Wave	−.001	(.033)	.024	(.030)	−.081^*^	(.033)	−.076^*^	(.031)
Acceptable to Break Moral Rules	.175^***^	(.016)	.176^***^	(.016)	.173^***^	(.015)	.173^***^	(.014)
Age	−.022^**^	(.008)	−.025^**^	(.008)	.006	(.008)	.004	(.007)
Religiosity	−.132^*^	(.060)	−.133^*^	(.060)	−.107^*^	(.046)	−.110^*^	(.046)
Moral Relativism	.321^***^	(.021)	.320^***^	(.021)				
Moral Progressivism					.322^***^	(.020)	.321^***^	(.020)
Constant	.130^*^	(.060)	.060	(.048)	.240^***^	(.061)	.231^***^	(.049)

*Note: N* = 3,231; Obsv. = 9,693.

* *p* < .05; **p < .01; ***p < .001 (two-tailed tests).

Analyses based on random-effects estimation are presented as Models 2 and 4 in Table 1.^6^ For moral progressivism, majoring in HASS fields and pursuing graduate studies are the most reliable predictors. These effects are positive, meaning these students are more likely to believe people should adjust their moral beliefs to reflect social change. Majoring in HASS is also an important predictor of moral relativism, but we also find a significant effect for STEM majors. Unlike moral progressivism, however, the effects on moral relativism are negative, suggesting HASS and STEM majors are significantly *more* likely than students not enrolled in higher education to believe there are definite rights and wrongs.^7^

**Figure 2.**
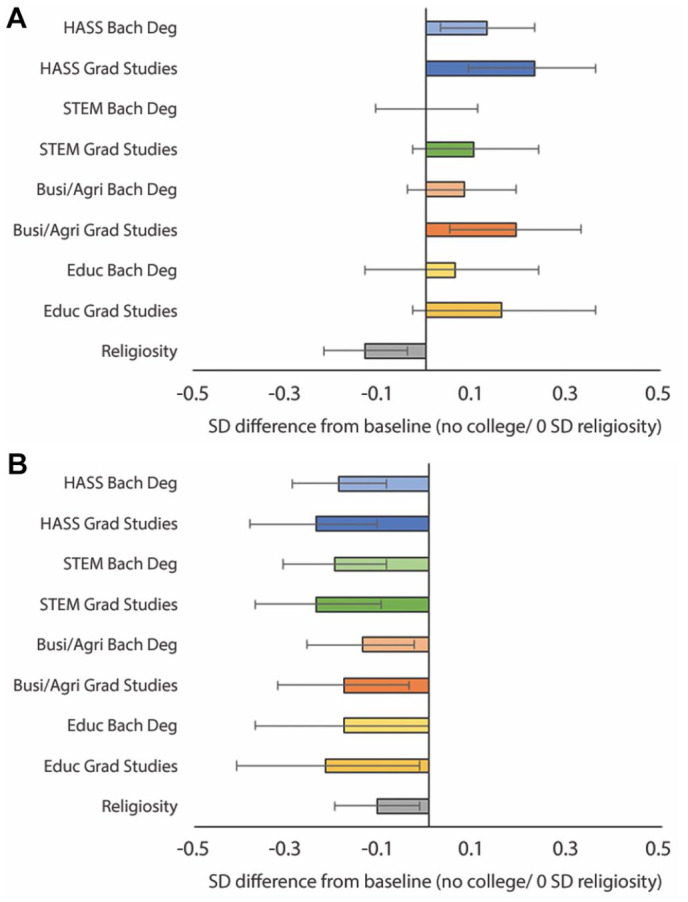
Panel A: Predicted Moral Progressivism across Higher Education / Predicted Value for 1 SD Religiosity; Panel B: Predicted Moral Relativism across Higher Education / Predicted Value for 1 SD Religiosity *Note*: 95 percent confidence intervals.

Of course, fields of study and educational attainment effects are rarely observed in isolation from one another. To give a better sense of their cumulative effects, we plot predicted levels of moral progressivism and moral relativism across majors for individuals with a bachelor’s degree or pursuing graduate studies compared to those who never enrolled in college. We set the wave variable to 4 (the last wave) and hold all other variables at their sample means. Results are shown in Figure 2, along with 95 percent confidence intervals (shown in the text in brackets). Consistent with previous literature, higher education predicts greater moral progressivism. This effect is strongest among HASS majors, for whom finishing a bachelor’s degree or pursuing graduate studies is associated with .13 SD [.02, .24] and .23 SD [.10, .36] increases, respectively, in moral progressivism compared to individuals who never attend college. Higher moral progressivism is also evident among business/agriculture majors (.19 SD [.04, .33]), but only if they pursue graduate training. The lower bounds of the 95 percent confidence intervals show that, conservatively, true cumulative effects might be quite small. However, the noticeable outlier is the lower-bound effect of HASS majors who enter graduate programs—here, the lower-bound effect of .10 SD is more than twice as large as the next largest lower bound (.04 SD, for business majors).

Turning to predictions for moral relativism, Figure 2 shows that getting a bachelor’s degree in any field except education predicts lower moral relativism compared to individuals who do not enroll. This effect grows among people pursuing graduate studies, with moral relativism being lower for students in all fields, suggesting a general effect of higher education. However, the precision of these predictions varies across educational categories. Examining the upper-bounds of the 95 percent confidence intervals (upper bounds because effects are negative) shows that, conservatively, the decrease in moral relativism is likely to be greatest among HASS majors (–.10 SD) and STEM majors (–.11 SD) who pursue graduate studies. Degrees in business and education also predict lower moral relativism, but the effects are less precise, with upper-bounds of the 95 percent confidence intervals approaching 0.

Of course, the fact that moral change seems to occur does not necessarily imply it is substantively meaningful. One way to assess this is by comparing the effects for higher education to the effects of other social institutions understood as intimately related to morality. We do so by considering how the effect compares to religiosity—a scale capturing the intensity of both religious beliefs and practice. Predicted values for effects of a 1 SD increase in religiosity are plotted in Figure 2. Substantively, a 1 SD difference in religiosity roughly amounts to the difference between someone who is actively religious and someone who is not religious.^8^ Each SD increase in religiosity is associated with a –.13 SD [–.25, –.02] decrease in moral progressivism, which is roughly half the size of the largest predicted educational effects on moral progressivism, although in the opposite direction. Like higher education, however, a 1 SD increase in religiosity is associated with a decrease in moral relativism (–.11 SD [–.20, –.02]), but again it is about half the size of higher education’s stronger effects. It follows that the effect of higher education on moral attitudes can rival and even offset the effect of adolescent religiosity. This is striking given the putative focus in higher education on knowledge, compared to the explicit moral agenda of organized religion.

### Moral Concern for Others, Moral Concern for Social Order, and Higher Education

Our analysis suggests that pursuing higher education—particularly in HASS degrees or at the graduate level—promotes a moral profile characterized by a progressive belief that morals should be adapted to changing societal needs accompanied by a conviction that there are definite moral truths. The content of those moral truths, however, remains unclear. To address this, we now turn to higher education’s effect on moral concerns for others and for social order.

In contrast to the previous analyses, concern for others and for social order were only measured at wave 4. This is good news for issues of temporal ordering—educational decisions necessarily precede measurement of the two moral attitudes—but it also means we cannot test for and, as needed, use fixed-effects estimation to control for omitted confounds. Consequently, the adequacy of our estimates depends more heavily on the level of confounding and the ability of our control variables to adjust for it. We are encouraged by the fact that fixed-effects estimation was not needed for the previous analyses, which suggests confounding from time-invariant predictors might also be relatively low in the present analyses. To be cautious, we still control for variables tied to college enrollment, educational attainment, major choice, and sample attrition (see Appendix C). However, we cannot eliminate the possibility that some relevant controls have been omitted.

**Figure 3.**
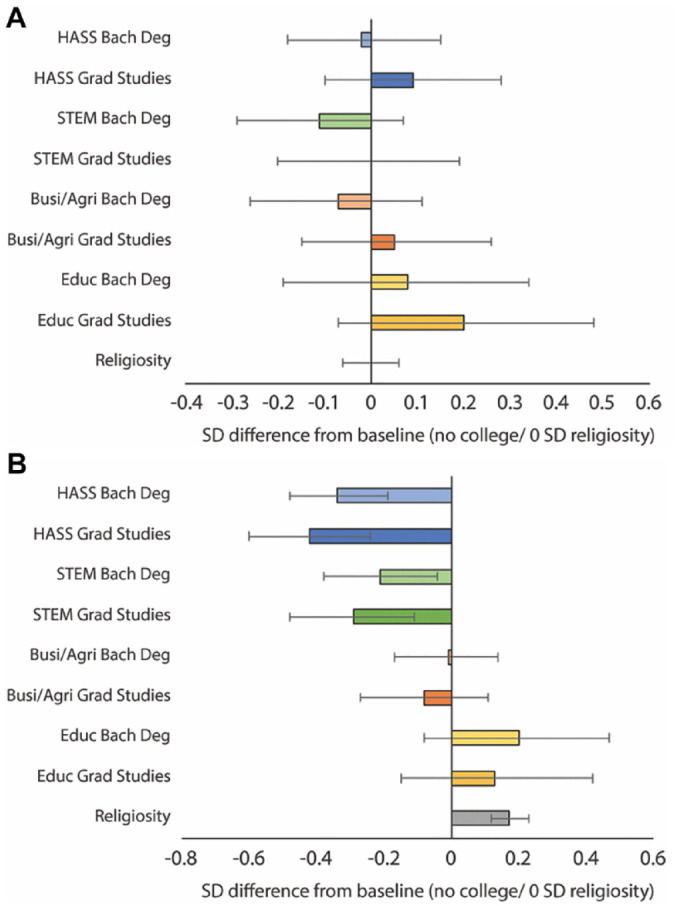
Panel A: Predicted Moral Concern for Others across Higher Education / Predicted Value for 1 SD Religiosity; Panel B: Predicted Moral Concern for Social Order across Higher Education / Predicted Value for 1 SD Religiosity *Note*: 95 percent confidence intervals.

Figure 3 displays the key results of linear models assessing how educational attainment and fields of study predict moral concerns for others and for social order (full results are in Appendix Table D2). Bars in Figure 3 represent the predicted differences in moral concern for others and for social order for each educational outcome compared to respondents with no college, with all other values set to the mean. There are a few notable patterns. First, educational attainment does not appear to influence moral concern for others—everyone, it seems, endorses principles of care and justice, and this does not change much while pursuing higher education.^9^ The story is different for concern for social order. Educational attainment appears to progressively diminish concern for social order, with each increase in attainment corresponding with lower concern. Results also vary by field of study. For students majoring in business/agriculture or education, moral concern for social order is not significantly different from those with no college experience. By contrast, HASS and STEM majors experience significant changes. These effects are especially pronounced for students majoring in HASS: attaining a bachelor’s degree in these fields is associated with a –.34 SD [–.49, –.20] decrease in concern for order compared to individuals who never enroll in higher education. The effect increases to –.42 SD [–.60, –.24] for students who continue on to graduate studies.

To assess how meaningful these effects are, we again compare them to the effects of adolescent religiosity. Like higher education, religiosity significantly predicts moral concern for social order but not moral concern for others. A 1 SD increase in religiosity corresponds to a .17 SD [.11, .22] increase in moral concern for social order. This effect is about one half the size of the effect of obtaining a bachelor’s degree in a HASS field but in the opposite direction. Given that this effect may be partly suppressed by the controls for adolescent religious affiliation already in the model, we also consider this effect in tandem with religious denomination. For instance, Evangelical Christians (the largest religious denomination in our survey) that are 1 SD higher on religiosity are predicted to have moral concern for social order that is .38 SD [.23, .53] higher than non-religious teens, holding all other variables at their means. This is slightly larger than the effect of obtaining a bachelor’s degree in HASS (–.34 SD [–.49, –.20]), but slightly smaller than the effect for HASS majors who go on to pursue a graduate degree (–.42 SD [–.60, –.24]). As with the prior analyses of moral progressivism and relativism, higher education can have an effect on moral concern for social order that rivals the influence of religious exposure and involvement during the teenage years.

Our analysis underscores higher education’s importance for moral socialization. We find notable effects across fields of study, and HASS degrees in particular have strong effects on moral relativism, progressivism, and concern for social order. By increasing moral progressivism and decreasing concern for social order, moral change in HASS degrees direct students toward stereotypical liberal moral profiles. However, the *decrease* in moral relativism promoted by these degrees denotes a departure from typical liberal morality. This suggests HASS majors—and to a lesser degree many other college-educated students—differ from the common liberal moral profile because of their *absolutist* assertion of similar moral sensibilities.

### Assessing the Scope of Higher Education’s Effects

We assess the scope of higher education’s effects in two ways. First, we focus on HASS effects and examine whether these effects are consistent across students whose parents hold different political ideologies. This examines the possibility that moral changes are concentrated in particular ideological groups that may be more or less predisposed to accept liberal moral claims. To test this, we interact majoring in HASS by the ideological identification of respondents’ parents during wave 1.^10^ Because interactions lead to smaller numbers of cases in each category of educational attainment, our analysis combines individuals with bachelor’s degrees and graduate studies in an effort to maintain statistical power. We focus on HASS given that the previous analyses found this major to have the most consistent effects.

**Figure 4.**
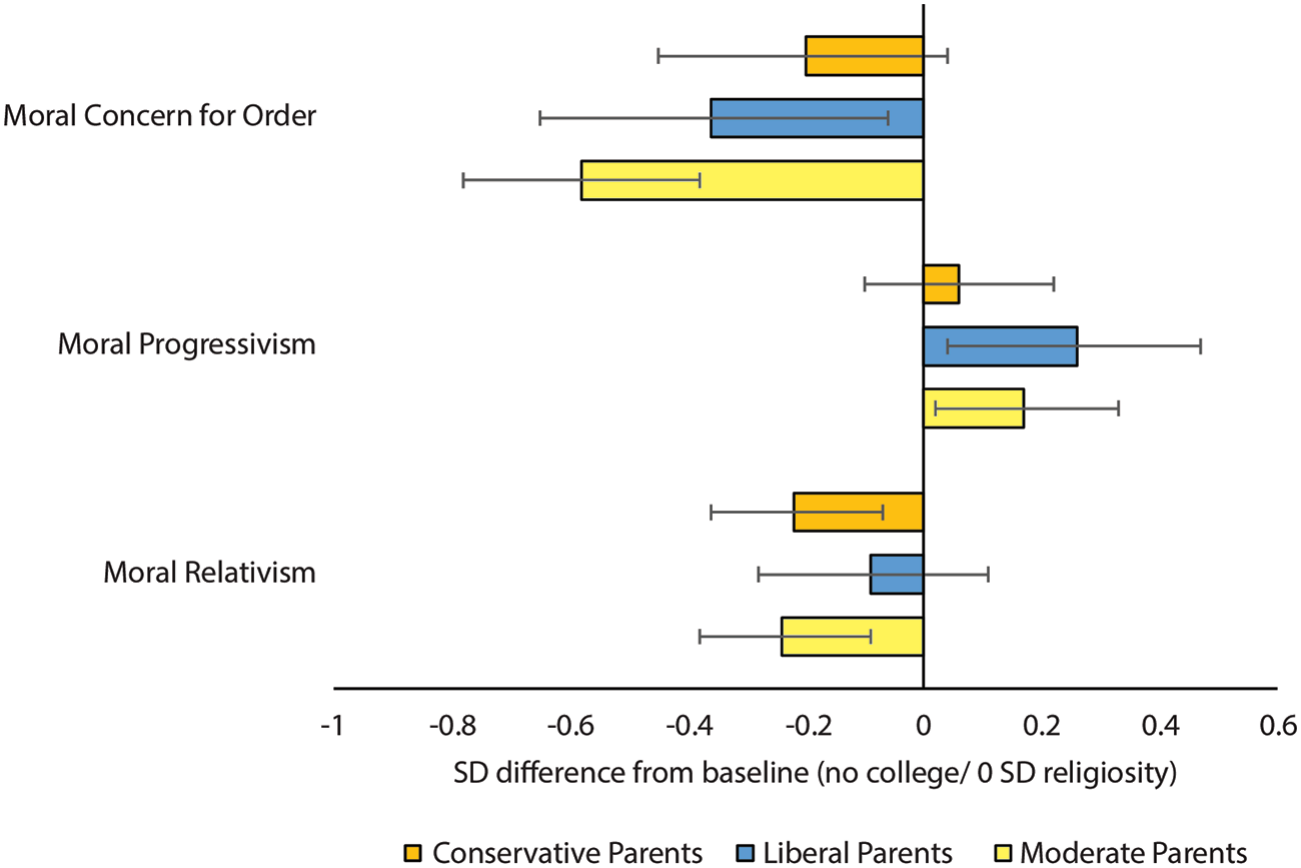
Predicted Moral Relativism, Moral Progressivism, and Concern for Social Order for Degree-Holders in HASS across Parental Ideology, Compared to Respondents Not Enrolled in Higher Education with Respective Parental Ideology *Note*: 95 percent confidence intervals.

Figure 4 plots HASS effects for students with conservative, moderate, and liberal parents. Bars denote differences (in SD units) for HASS majors compared to respondents without higher education from households with the same ideological leanings (for the full model, see Appendix Table D3). The effects of majoring in HASS on moral relativism are non-significant for students from liberal households, as are the effects on moral progressivism and moral concern for social order for students from conservative households. This might indicate that parental influence partly shields students from moral change. On the other hand, the pattern of effects is the same across all subgroups, leaving open the possibility that non-significant effects simply reflect insufficient statistical power. And some change is evident by conventional standards among students from all ideological backgrounds. Change is most pronounced among students from moderate households, perhaps owing to their lack of prior ideological commitments. These students experience change on all three variables, with our models predicting increased endorsement of moral progressivism (.17 SD [.01, .32]), decreased concern for social order (–.58 SD [–.78, –.38]), and a growing sense of certainty that definite rights and wrongs exist (–.24 SD [–.39, –.10]). The fact that the patterns of change are generally consistent across students from differing ideological backgrounds, and strongest among those from moderate homes, indicates the effects of a HASS degree are not restricted to students who are predisposed toward liberal morality.

The second way we examine the scope of higher education’s effects is by examining different hypothetical educational paths and exploring their effects on moral attitudes. We use the models from Table 1 and Appendix Table D2 to calculate predicted effects on moral progressivism, moral relativism, and moral concern for social order for different combinations of educational attainment and fields of study, holding all other variables at their means. Predicted effects represent differences between pathway-specific predictions and the predicted value for individuals with no college for each moral dimension.

Results are displayed in Table 2. The first line gives the predicted moral progressivism, moral relativism, and concern for social order for a hypothetical average individual who does not enroll in higher education. Our models predict this person would score –.02 SD below the sample average in moral progressivism, –.03 SD below the average on moral relativism, and .13 above the average on concern for social order. Statistically significant differences from this baseline are indicated with asterisks in Table 2. For example, the moral progressivism of HASS majors enrolled in graduate studies is predicted to be .23 SD higher than the predicted value for respondents not in college, with this difference reaching statistical significance.

**Table 2.** Moral Outcomes across Different Educational Trajectories

			Moral Progressivism		Moral Relativism		Social Order		Any Shift toward Liberal Moral Profile?
Field	Attainment	% of Enrolled Respondents	Predicted Value	Direction of Change	Predicted Value	Direction of Change	Predicted Value	Direction of Change	
	No College		−.018 (ref.)		−.027 (ref)		.122 (ref.)		
HASS	Some College	9.6%	.122^***^	liberal	−.126^***^	unclear	−.270^***^	liberal	yes
	Bachelor’s	21.4%	.130^*^	liberal	−.192^***^	unclear	−.343^***^	liberal	yes
	Graduate	9.4%	.232^***^	liberal	−.236^***^	unclear	−.418^***^	liberal	yes
STEM	Some College	10.9%	−.009		−.132^***^	unclear	−.140		no
	Bachelor’s	11.6%	−.001		−.197^***^	unclear	−.213^*^	liberal	yes
	Graduate	7.5%	.101		−.242^***^	unclear	−.288^**^	liberal	yes
Business/Agriculture	Some College	6.4%	.076		−.071		.066		no
	Bachelor’s	16.1%	.083		−.136^**^	unclear	−.008		no
	Graduate	3.6%	.186^**^	liberal	−.181^**^	unclear	−.082		yes
Education	Some College	0%	.049		−.112		.275		no
	Bachelor’s	2.0%	.056		−.177		.202		no
	Graduate	3.5%	.159		−.222^*^	unclear	.127		no

*Note*: Predicted values and significance tests compare difference from no college in SD units for each moral dimension. The “direction of change” column refers to whether the difference from no college is in the direction of a more stereotypically liberal/conservative moral profile. Percentages of enrolled respondents were calculated at wave 4 using listwise deletion; the total exceeds 100 percent because of double majors.

* *p* < .05; ^**^p < .01; ^***^p < .001 (two-tailed tests).

Table 2 makes it clear that most educational pathways are expected to shift at least one of the three moral attitudes. In most cases, the changes are toward a stereotypically liberal moral profile—that is, toward higher levels of moral progressivism or lower concern for social order. Higher education also consistently predicts lower moral relativism, but here the relationship to liberal and conservative morality is less clear. As recent academic writing about victimhood culture and related phenomena make clear, moral certainty may be a feature of both liberal and conservative moral attitudes, so a reduction in moral relativism could signal change toward either profile. Still, the predominant pattern is toward greater endorsement of liberal morality. Compared to individuals who never enroll in higher education, all degree-holders in HASS or STEM fields are expected to shift toward liberal morality in some way. Among business, agriculture, and education majors, one of six pathways is predicted to have a liberalizing effect, three show no significant differences from those not in college, and two influence only moral relativism and hence their relationship to liberal and conservative morality is unclear. If we consider the distribution of educational pathways in our sample, this indicates that higher education will liberalize morality for about 63 percent of students, have an unclear effect for 30.5 percent of students, and have no discernable effect for 8.4 percent of students. Due to sampling variability and the small sample sizes for some of the educational pathways, these predictions should be viewed only as rough estimates—however, it is clear that for the majority of students, higher education is likely to be a morally liberalizing experience.

## Discussion

According to Bloom (1987:26), behind the curriculum of every educational system lies a latent moral purpose to “produce a certain kind of human being.” Yet recent scholarship has questioned whether the collegiate experience is indeed a deeply formative period. Researchers have demonstrated that differences prior to enrollment explain much of the variation in outcomes across educational levels (Campbell and Horowitz 2016; Elchardus and Spruyt 2009; Gross 2013), a finding that resonates with work emphasizing the importance of early-life social experiences in forming moral dispositions (Killen and Smetana 2015; Vaisey and Lizardo 2016). We test whether higher education shapes morality using four waves of data that follow respondents from high school into young adulthood and models that test or control for selection processes. We find that moral attitudes remain malleable into young adulthood and that higher education is an important institution that facilitates change.

The most consistent predictors of moral change were pursuing graduate education and majoring in the humanities, arts, or social sciences. These educational experiences increased belief that moral principles should adapt to changes in society (moral progressivism), but—in contrast to the typical liberal moral profile—they also *decreased* moral relativism, suggesting some students are emerging from higher education with a greater conviction in absolute rights and wrongs. However, our data indicate this moral absolutism looks different than the moral absolutism of religious and political conservatives. Rather than supporting traditional norms, these students emerge from university with a moral profile characterized by high concern for others and weak commitment to traditional social order. One interpretation of these results is that some university students—particularly those majoring in HASS or who continue on to graduate education—come to believe that the morals of society must change to remedy historical (and current) injustices (i.e., moral progressivism), but that the moral principles they have learned through their studies represent the *real* moral truth (moral absolutism).

Evidence of decreased relativism is noteworthy in that it contrasts with prior critiques of higher education by religious and conservative commentators, as well as earlier scholarly accounts that described relativistic tendencies among academics (Hunter 1991; Wuthnow 1988). Lazarsfeld and Thielens’s (1958) pioneering study of the U.S. professoriate, for instance, described social scientists as relativists whose keen awareness of historical variation in morality led to contingency in their own beliefs. Consistent with this, we find HASS majors believe morals should be adjusted to social changes, suggesting a more contextual and relativistic moral understanding. However, these students differ from earlier relativists in their willingness to claim there are definite moral truths. This lends prima facie support to recent claims that the moral relativism of years past is transforming into a form of liberal moral puritanism (Campbell and Manning 2018; Lukianoff and Haidt 2018).

The apparent discrepancies between our findings and earlier work invite the question of whether key socializing processes in higher education have changed. Our study’s focus on individual-level change limits our ability to assess this directly, but suggestive research allows us to speculate. Growing social closure along the lines of political ideology among university faculty and administrators may partly explain the rise in moral absolutism among students (Gross 2013). In 1969, 28 percent of professors described themselves as conservative, but by 2013 this decreased to 12 percent (Eagan et al. 2014; Ladd and Lipset 1975). Data on college administrators are harder to come by, but a recent survey found that among “student-facing” college administrators—those who are most responsible for shaping student experiences on campus—liberals outnumber conservatives by as much as 12 to 1 (Abrams 2018a, 2018b). Increasing political homogeneity among faculty and/or administrators could create a sense of moral consensus that leaves shared liberal beliefs unchallenged or might even make them seem naturally true. Lack of interpersonal engagement with members of an outgroup can in turn make individuals less politically tolerant, less likely to regard opposing views as legitimate, and more likely to hold extreme attitudes (Huckfeldt, Mendez, and Osborn 2004; Mutz 2002)—all traits that coincide with stronger moral conviction (Skitka et al. 2021). These processes could contribute to a sense of liberal moral certitude among students to the extent that university messaging, course content, the types of faculty mentors available, or even informal interactions with faculty and staff communicate moral consensus.

This narrative may be incomplete, however, given that moral certainty also increases for students enrolled in majors that are not heavily associated with liberal moral concerns.^11^ Another possibility is that growth in moral certainty might also be explained by socialization into the official culture of dominant institutions. According to scholarship in this area, universities are the primary institution for mobility into the professional classes. Consequently, their latent function is to socialize students into dominant status culture by teaching proper etiquette, aesthetic tastes, and moral evaluations that serve to legitimize their advantaged class position (Bourdieu 1984; Collins 1971; Jackman and Muha 1984). Moral justifications may differ across fields, with educated elites variously casting themselves as “enlightened cosmopolitans” (see Johnston and Baumann 2007; Lizardo and Skiles 2015; Ollivier 2008) or winners of “meritocratic struggle” (Bourdieu and Passeron 1979; Mijs 2016; Piketty 2020), but strong moral self-assurance appears to form a common sentiment. Importantly, as cultivation combines with a growing sense of expertise from formal training, educational attainment may impart moral beliefs with a stamp of objectivity (cf. Bottum 2014). Seen this way, moral righteousness might be a consequence of rising social class rather than liberal socialization alone. Of course, the two need not be mutually exclusive—professionalization and liberal attitudes could reinforce one another to the extent that dominant institutions adopt liberal values, policies, or agendas. Some evidence suggests this process might be well under way.^12^

Recent events suggest higher education’s role in liberalizing moral concerns could have important consequences for social conflict. Scholars have noted the growing salience of the “diploma divide” in politics, with educational attainment being among the strongest predictors of voting against Donald Trump, Brexit, and other events (Gidron and Hall 2017; Lind 2020; Piketty 2020). Our study speaks to the moral dimension of this divide. When conflict pits nativism against cosmopolitanism and “vulgar” populism against “technocratic” expertise, an educational system that promotes commitment to liberal sensibilities will likely stratify voters according to educational attainment.^13^ Moral stratification of this sort could pose several risks to civil society. If individuals on the political right come to regard the primary credentialing institution as hostile to their interests, partisan segregation could further escalate by deterring conservative enrollment (Gross 2013). This, in turn, could deepen the distrust toward government, media, and other institutions that employ the credentialled classes that is already evident among the less-educated (Rainie and Perrin 2019). Finally, deliberative democracy could suffer if educational attainment is accompanied by a rising moral conviction that views opposition as too dangerous to engage with or even tolerate (Skitka 2010; Skitka, Bauman, and Sargis 2005).^14^

However, we must be careful not to overstate the political consequences of moral change. Partisans often differ in their moral attitudes (Miles and Vaisey 2015), but it is unclear whether higher education’s effects on moral attitudes will necessarily lead to demonstrable shifts in political behavior. A student leaving the university might well emerge with less regard for traditional conservative morality, yet still vote Republican for economic, foreign policy, or other reasons. Some research even finds that partisan identification precedes moral change, suggesting moral differences may *express* rather than *constitute* partisan allegiances (Hatemi, Crabtree, and Smith 2019; Smith et al. 2017). The fact that higher education also shapes eventual class position complicates matters further by leaving open the possibility that material interests underlie conflict that on the surface appears morally motivated (Lasch 1994; Lind 2020; Piketty 2020). Given these considerations, it would be premature to conclude that morality is the only or even necessarily the primary predictor of political behavior. Future research should continue to explore how moral, economic, and political interests intersect among the highly educated, and the effects these have on political behavior. Such research could build on older sociological analyses of the “New Class” emerging from the knowledge economy (Bazelon 1967; Bell 1979; Gouldner 1978), variously treated as the “Creative Class” (Florida 2002), the “Elect” (Bottum 2014), or the “Brahmin Left” (Piketty 2020) in contemporary discussions.

Our study also speaks to work on moral socialization (Guhin, Calarco, and Miller-Idriss 2021). Contrary to recent accounts emphasizing selection effects, we find that moral socialization occurs within universities in a meaningful way. Consider higher education’s effect as it compares to religious practices. Scholars often depict religion as the defining cleavage of cultural conflict (Castle 2019; Gorski 2020; Wuthnow 1989), yet our analysis finds that the effect of higher education on moral concerns is comparable to the moral influence of adolescent religion and imparts a sense of moral absolutism that rivals the effect of religiosity. Evidence of moral change invites additional research into what aspects of early morality are stable, and which are open to revision. Theories of moral socialization often acknowledge the possibility of later moral change, but in practice focus on innate moral impulses or moral learning processes that occur early in life (Graham et al. 2009; Killen and Smetana 2015). Scholars who consider attitude development during adulthood, moreover, find greater support for a “settled disposition model” emphasizing stability rather than change (Kiley and Vaisey 2020; Vaisey and Lizardo 2016). However, our results suggest adolescence and young adulthood remain important periods of moral change worthy of scholarly attention (cf. Hardy and Carlo 2011).

Further work is also needed to understand the processes whereby educational attainment influences moral attitudes. Consistent with the socialization hypothesis, moral change was strongest for HASS students, and comparatively weaker and in some cases absent for other majors. This suggests curricular content matters for moral change. The traditional socialization hypothesis holds that moral relativism is the natural by-product of exposure to cultural diversity, but this was not borne out by our analyses. Instead, we observed an increase in moral absolutism, which may suggest students are being actively taught moral ideals. This, however, remains speculative and requires systematic exploration. Furthermore, the fact that moral relativism decreases across all fields suggests socialization effects likely are not due to curricular content alone and may indicate social learning through noncurricular aspects of the university experience. As discussed earlier, we speculate that formal and informal socialization into official culture might explain this effect, with institutional validation and expertise giving students moral self-assurance, and the mostly liberal direction of this change signaling the elevation of social justice and related liberal concerns within major institutions (Campbell and Manning 2018; Lind 2020).

Ideally, future research would address the limitations of this study. For example, future work should use larger samples to increase statistical power to detect effects when cross-classifying educational categories. Furthermore, we believe our research supports a causal interpretation, but this interpretation is necessarily provisional, particularly for our results linking higher education to changing moral concerns for order, given that these were measured only at wave 4. Researchers should collect data on moral concerns at multiple waves so that correlated-random-effects models or equivalent methods can be used to test for and—if needed—correct for the influence of unobserved time-constant confounds. Future analysis could also unpack the causal mechanisms involved by incorporating direct measures of course content and noncurricular aspects of the academic environment (e.g., campus messaging, programming, friendship networks; see Rauf 2021; Strother et al. 2020). The moral consequences of cognitive sophistication could also be clarified. Indeed, absolute moral certitude appears at odds with the cognitive hypothesis, which predicts greater intellectual flexibility as a result of sophistication (cf. Adorno et al. 1950; Altemeyer 1996; Jost et al. 2003). Finally, it is important to replicate our results using recent samples of college-aged adults. Although victimhood culture (under various names) has been discussed since at least the 1980s (Bloom 1987), some scholars argue that manifestations of this moral culture increased sharply beginning in the mid-2010s (Campell and Manning 2018; Lukianoff and Haidt 2018). The final wave of data for the NSYR was collected in 2012 to 2013, which places our data relatively early in these developments. More recent data would allow our findings to be tested in a sample that more closely aligns with the theorized timeline and could provide important insights into the underlying mechanisms.

## Appendix

### Part A: Measurements and Coding

#### Moral Attitudes

##### Moral Concern for Others (Using the 20-Item Moral Foundation Questionnaire)

Scores were derived by averaging responses for the eight questions related to care and fairness. The Cronbach alpha for the scale was α = .71.

###### Care/Harm

How much do you agree with the following?

Compassion for those who are suffering is the most crucial virtue.

One of the worst things a person can do is hurt a defenseless animal.

(1 = strongly disagree, 2 = moderately disagree, 3 = slightly disagree, 4 = slightly agree, 5 = moderately agree, 6 = strongly agree)

When you decide whether something is right or wrong, to what extent are the following considerations relevant to your thinking?

Whether or not someone suffered emotionally.

Whether or not someone cared for someone weak or vulnerable.

(1 = not at all relevant, 2 = not very relevant, 3 = slightly relevant, 4 = somewhat relevant, 5 = very relevant, 6 = extremely relevant)

###### Fairness/Cheating

How much do you agree with the following?

When the government makes laws, the number one principle should be ensuring that everyone is treated fairly.

Justice is the most important requirement for a society.

(1 = strongly disagree, 2 = moderately disagree, 3 = slightly disagree, 4 = slightly agree, 5 = moderately agree, 6 = strongly agree)

When you decide whether something is right or wrong, to what extent are the following considerations relevant to your thinking?

Whether or not some people were treated differently than others.

Whether or not someone acted unfairly.

(1 = not at all relevant, 2 = not very relevant, 3 = slightly relevant, 4 = somewhat relevant, 5 = very relevant, 6 = extremely relevant)

##### Moral Concern for Social Order (Using the 20-Item Moral Foundations Questionnaire)

Scores were derived by averaging responses for the 12 questions related to loyalty, authority, and sanctity. The Cronbach alpha for the scale was α = .79.

###### Loyalty/Betrayal

How much do you agree with the following?

I am proud of my country’s history.

People should be loyal to their family members, even when they have done something wrong.

(1 = strongly disagree, 2 = moderately disagree, 3 = slightly disagree, 4 = slightly agree, 5 = moderately agree, 6 = strongly agree)

When you decide whether something is right or wrong, to what extent are the following considerations relevant to your thinking?

Whether or not someone’s actions showed love for his or her country.

Whether or not someone did something to betray his or her group.

(1 = not at all relevant, 2 = not very relevant, 3 = slightly relevant, 4 = somewhat relevant, 5 = very relevant, 6 = extremely relevant)

###### Authority/Subversion

How much do you agree with the following?

Respect for authority is something all children need to learn.

Men and women each have different roles to play in society.

(1 = strongly disagree, 2 = moderately disagree, 3 = slightly disagree, 4 = slightly agree, 5 = moderately agree, 6 = strongly agree)

When you decide whether something is right or wrong, to what extent are the following considerations relevant to your thinking?

Whether or not someone showed a lack of respect for authority.

Whether or not someone conformed to the traditions of society.

(1 = not at all relevant, 2 = not very relevant, 3 = slightly relevant, 4 = somewhat relevant, 5 = very relevant, 6 = extremely relevant)

###### Purity/Degradation

How much do you agree with the following?

People should not do things that are disgusting, even if no one is harmed.

I would call some acts wrong on the grounds that they are unnatural.

(1 = strongly disagree, 2 = moderately disagree, 3 = slightly disagree, 4 = slightly agree, 5 = moderately agree, 6 = strongly agree)

When you decide whether something is right or wrong, to what extent are the following considerations relevant to your thinking?

Whether or not someone violated standards of purity and decency.

Whether or not someone did something disgusting.

(1 = not at all relevant, 2 = not very relevant, 3 = slightly relevant, 4 = somewhat relevant, 5 = very relevant, 6 = extremely relevant)

##### Moral Progressivism

Some people say that the world is always changing and we should adjust our views of what is morally right and wrong to reflect those changes.

(1 = strongly disagree, 2 = disagree, 3 = agree, 4 = strongly agree)

##### Moral Relativism

Some people say that morals are relative, that there are no definite rights and wrongs for everybody.

(1 = strongly disagree, 2 = disagree, 3 = agree, 4 = strongly agree)

#### Control Variables

*Parental income*: (1 = less than $10K, 2 = $10K to $20K, 3 = $20K to $30K, 4 = $30K to $40K, 5 = $40K to $50K, 6 = $50K to $60K, 7 = $60K to $70K, 8 = $70K to $80K, 9 = $80K to $90K, 10 = $90K to $100K, 11 = more than $100K).

*Parental education*: 0 = no college, 1 = either parent completed at least some college.

*Household size*: Total number of people in household measured as a continuous variable.

*Parent immigrant*: 0 = interviewed parent was born with U.S. citizenship, 1 = parent being interviewed was born without U.S. citizenship.

*Race*: Black, Hispanic, Asian measured as dummy variables. White/other as reference group.

*Religion*: Mainline Protestant, Catholic, Evangelical Protestant, African American Protestant, Jewish, Mormon, other religion measured as dummy variables, with non-religious as the reference category.

*Parental ideology*: Conservative parent (parent reports being somewhat conservative or very conservative), liberal parent (parent reports being somewhat liberal or very liberal), measured as dummy variables, with moderates being reference category.

*Gender*: 0 = male, 1 = female.

*Importance of child graduating college for parents*: Measured as ordinal variable with categories, 1 = not at all important, 2 = not very important, 3 = somewhat important, 4 = very important, 5 = extremely important.

*High school grades*: 1 = mostly Fs, 2 = Ds and Fs, 3 = mostly Ds, 4 = Cs and Ds, 5 = mostly Cs, 6 = Bs and Cs, 7 = mostly Bs, 8 = As and Bs, 9 = mostly As, 10 = all As. If student reported “mixed” grades, counted as missing.

*College aspirations*: 0 = respondent does not aspire to finish college degree, 1 = respondent would like to be a college graduate or pursue postgraduate degree.

*Popularity at school*: How much would you say you are part of the popular group at school? (1 = none, 2 = a little, 3 = some, 4 = a lot).

*Feelings of meaninglessness*: How often, if ever, does life feel meaningless to you? (1 = never, 2 = rarely, 3 = sometimes, 4 = usually, 5 = always).

*Conformity*: How important or unimportant is it to you to fit in with what teens your age think is cool? (1 = not important at all, 2 = not very important, 3 = somewhat important, 4 = very important, 5 = extremely important).

##### Social Concern

Scale ranging from 1 to 4 (from less to more care), composed of three questions:

How much do you personally care or not about equality between different racial groups?

How much do you personally care or not about the needs of poor people in this country?

How much do you personally care or not about the needs of elderly in this country?

(1 = not really care, 2 = care a little, 3 = care somewhat, 4 = care very much)

The Cronbach score for this scale is α = .55.

##### Religiosity Composite Score

Scores were derived from combining all items from Pearce, Hayward, and Pearlman’s (2017) five-factor model into a single latent religiosity composite score.

###### Religious Belief

Do you believe that there is life after death? (0 = not at all/maybe; 1 = definitely)

Do you believe in the existence of angels? (0 = not at all/maybe; 1 = definitely)

Do you believe in the existence of demons or evil spirits? (0 = not at all/maybe; 1 = definitely)

Do you believe in the possibility of divine miracles from God? (0 = not at all/maybe; 1 = definitely)

Do you believe that there will come a judgment day when God will reward some and punish others, or not? (0 = not at all/maybe; 1 = definitely)

###### Religious Exclusivity

Is it okay for religious people to try to convert other people to their faith, or should everyone leave everyone else alone? (0 = leave others alone; 1 = okay)

Do you think it is okay for someone of your religion to also practice other religions, or should people only practice one religion? (0 = okay to practice other religions; 1 = should only practice one religion)

Do you think it is okay for someone of one religion to also practice other religions, or should people only practice one religion? (0 = okay to practice other religions; 1 = should only practice one religion)

Which of the following statements comes closest to your own views about religion? (0 = many religions may be true/there is very little truth in any religion; 1 = only one religion is true)

Some people think that it is okay to pick and choose their religious beliefs without having to accept the teachings of their religious faith as a whole. Do you agree or disagree? (0 = disagree; 1 = agree)

###### External Practice

How often do you attend church? (0 = never, 1 = few times a year, 2 = many times a year, 3 = once a month, 4 = 2 to 3 times a month, 5 = once a week, 6 = more than once a week)

In the last year, have you prayed out loud or silently together with one or both of your parents, other than at meal times or at religious services? (0 = no; 1 = yes)

In the last year, have you shared your own religious faith with someone else not of your faith? (0 = no; 1 = yes)

In the last year, have you been a part of a religious support or evangelism or prayer group that meets at school? (0 = no; 1 = yes)

In the last year, have you been a part of any other scriptures study or prayer group? (0 = no; 1 = yes)

Does your Church have an organized youth group for teenagers, or not? (0 = no; 1 = yes)

###### Personal Practice

How often, if ever, do you pray by yourself alone? (0 = never; 1 = less than once a month; 2 = one to two times a month; 3 = about once a week; 4 = a few times a week; 5 = about once a day; 6 = many times a day)

How often, if ever, do you read from scriptures to yourself alone? (0 = never; 1 = less than once a month; 2 = one to two times a month; 3 = about once a week; 4 = a few times a week; 5 = about once a day; 6 = many times a day)

In the last year, have you fasted or denied yourself something as a spiritual discipline? (0 = no; 1 = yes)

In the last year, have you tried to practice a weekly day of rest to keep the Sabbath? (0 = no; 1 = yes)

###### Religious Salience

If you were unsure of what was right or wrong in a particular situation, how would you decide what to do? Would you MOST likely: (0 = make me feel happy/help me get ahead/follow advice of adult/something else; 1 = God or Scripture says is right)

Have you ever made a personal commitment to live your life for God? (0 = no; 1 = yes)

How important or unimportant is religious faith in shaping how you live your daily life?

(0 = not at all important, 1 = very unimportant, 2 = somewhat important, 3 = very important, 4 = extremely important)

##### Attitudes toward Premarital Sex

Do you think that people should wait to have sex until they are married, or not necessarily? (1 = no; 2 = not necessarily; 3 = yes).

##### Acceptability of Breaking Moral Rules

Some people believe that it is sometimes okay to break moral rules if it works to your advantage and you can get away with it.

(1 = strongly disagree, 2 = disagree, 3 = agree, 4 = strongly agree)

#### Selection Into Wave 4

Controls for selection into wave 4 were selected by running iterative logistic regression models and assessing which wave 1 variables predicted participation at wave 4. We report only the variables that predicted selection into wave 4.

##### Ever Suspended in High School

In the last TWO years, how many times, if any, have you been suspended or expelled from school? (0 = never, 1 = >0)

##### Changed High School

How many times, if any, has [your teen] had to switch schools because of a residential move? (0 = never, 1 = >0)

##### Parent Homeowner

Do you own your current home, rent your current home, or have some other arrangement? (0 = do not own, 1 = own)

**Table A1.** Coding of Majors

**Humanities, Arts, Social Science** Art History History German Studies Liberal Arts General Studies Linguistics Spanish Studies English Literature French American Studies Classics Comparative Literature Religion Biblical Studies Philosophy Economics Criminal Justice Journalism Political Science Psychology Anthropology Sociology	**STEM** Biology Civil Engineering Geological Sciences Computer Science Zoology Biomedical Science Environmental Science Electrical Engineering Civil Engineering Mechanical Engineering Industrial Engineering Aerospace Engineering Chemistry Neuroscience Nursing Pharmacy Dental Hygiene Veterinary Technology Automotive Technologies Kinesiology Math Health Sciences
Geography Public Policy Child and Family Studies Government Social Work Child Development Human Development Information Systems Human Services Fine Art Photography Graphic Design Interior Design Architecture Theatre Music Music Education Media Studies Dance Film Vocal Performance **Education** Elementary Education History Education Secondary Education Childhood Education Early Childhood Education Teacher Education Physical Education Music Education	Physics Animal Science Speech, Language, and Hearing Sciences Audiology Microbiology Physical Education Physiology Nutrition Exercise Science **Business/Agriculture** Finance Business Administration Business Management Merchandizing Marketing Accounting Communication Entrepreneurship Project Management Advertising Public Relations Human Resources International Business Organizational Leadership Sport Management Agriculture Hospitality Management Operations Management

*Note*: Only majors that appeared more than once are listed in the table.

### Part B: Methodological Notes for Moral Progressivism and Moral Relativism Analyses

#### Model Specification

Linear models are widely used, widely understood, and easier to use with CRE analysis. Consequently, we used linear models for our analyses. However, given that our moral progressivism and moral relativism measures only have four response options, we tested whether using ordinal logistic regression would alter our conclusions. Results using ordinal models were substantively the same.

#### Fixed- versus Random-Effects Analyses

Here we delve deeper into why random-effects (RE) estimates might be preferred over fixed-effects (FE) estimates for the moral progressivism and relativism analyses. The primary reason is that some education variables—particularly those capturing field of study—have limited within-person variation across the three time points used in analyses. The amounts of within-person variation are shown in Table B1. Within-person variation for educational attainment variables is relatively sizeable, but for fields of study it is invariably low, and never exceeds 13 percent of the total variation in those variables. Additionally, coefficients for education variables are estimated holding other education variables constant, further reducing the amount of useable variation. The result is that FE estimates for education are often calculated using very little information. This increases standard errors, but it can also make estimates unreliable. The situation is analogous to estimating an effect in a model with high multicollinearity—the lack of unique information can lead to unusual or extreme estimated coefficients, and coefficients that change dramatically in response to small changes to the model.

**Table B1.** Between- and Within-Person Variance for Education Variables

	Between	Within	% Within
Some College	.150	.110	42.3
BA	.028	.052	65.0
Graduate	.014	.024	62.7
HASS	.097	.014	12.6
STEM	.075	.010	12.1
Business	.067	.009	11.5
Education	.014	.001	8.5

Given these considerations, using RE estimation may be preferable even at the risk of introducing some bias. The level of potential bias for a particular variable can sometimes be gauged by examining the coefficient for its cross-wave mean in the CRE model (see Table 1). As noted in the next section, larger coefficients suggest greater deviation from the FE estimates and hence potentially larger bias due to unobserved time-invariant confounds. However, these estimates become much less informative if the FE estimates are unreliable. In such cases, the cross-wave mean coefficients give the difference from FE estimates that might themselves be badly biased. In the present case, these considerations suggest FE estimation might be acceptable for educational attainment variables, whereas RE estimation might be more appropriate for the field of study variables given their low levels of within-person variation.

Fortunately, the basic pattern of results is consistent using both FE and RE estimation, giving us confidence that the main findings hold regardless of these modeling decisions. The major difference is in the size of the estimated effects, with the random-effects estimates generally being smaller in magnitude. The foregoing discussion suggests these smaller estimates might be more accurate, at least for the field of study variables. At a minimum, the RE coefficients give a conservative estimate of the size of education effects on moral attitudes.

#### Relationship between CRE and Hybrid Models

The correlated random-effects model (CRE; Wooldridge 2016) is

(1)
$$y_{it}=\mu_{t}+\beta_{CRE}x_{it}+\gamma_{CRE}{\bar{x}}_{i}+r_{i}+e_{it}$$

where *y* is an outcome that varies over individuals (*i*) and time (*t*). The *µ_t_* are intercepts that are allowed to vary at each time point. The *β_CRE_* are estimates of the time-varying variables (*x_it_*), and the *γ_CRE_* are the effects of the within-person, cross-time means of those variables (*x–_i_*). The error term consists of time-varying (*e_it_*) and time-invariant (*r_i_*) influences that are uncorrelated with the time-varying *x_it_*.

Allison (2009) presents a “hybrid” fixed/random-effects model that is quite similar:

(2)
$$y_{it}=\mu_{t}+\beta_{A}(x_{it}-{\bar{x}}_{i})+\gamma_{A}{\bar{x}}_{i}+r_{i}+e_{it}.$$

The major difference is that the time-varyingvariables are now deviated from their cross-wave means (*x_it_* – *x–_i_*). This provides a clear separation of within-person and between-person variation and allows the hybrid model to estimate both within-person effects (*β_A_*) and between-person effects (*γ_A_*).

We can rearrange Equation 2 to see how the hybrid model relates to the CRE. We multiply the *β_A_* through (*x_it_* – *x–_i_*), which gives us

$$y_{it}=\mu_{t}+\beta_{A}x_{it}-\beta_{A}{\bar{x}}_{i}+\gamma_{A}{\bar{x}}_{i}+r_{i}+e_{it}.$$

We then rearrange terms and factor out the cross-wave means (*x–_i_*):

(3)
$$y_{it}=\mu_{t}+\beta_{A}x_{it}+(\gamma_{A}-\beta_{A})\;{\bar{x}}_{i}+r_{i}+e_{it}.$$

Comparing Equation 3 to Equation 1 reveals the following equivalences:

$$\beta_{CRE}=\beta_{A}$$

$$\gamma_{CRE}=\gamma_{A}-\beta_{A}.$$

Because *β_A_* are estimated using only within-person variation, they provide fixed-effects estimates of the time-varying variables (*x_it_*). Because *β_CRE_* = *β_A_* it follows that the CRE also provides fixed-effects estimates.

Furthermore, the second equivalence shows the *γ_CRE_* estimate for the differences between the between-person (*γ_A_*) and within-person (*β_A_*) estimates of the time-varying variables. In the absence of time-invariant confounding with time-constant effects, the within- and between-person effects would be the same. It follows that testing whether the *γ_CRE_* are significantly different from 0 tests whether fixed-effects estimation is necessary.

#### Why Do Random- and Fixed-Effects Estimates (Sometimes) Differ So Much?

This section is intended mainly for readers wishing to better understand the changes in the coefficients for the education variables from the CRE to the random-effects model (Table 1 in the main text).

The CRE model is

$$y_{it}=\mu_{t}+\beta_{CRE}x_{it}+\gamma_{CRE}{\bar{x}}_{i}+r_{i}+e_{it}.$$

As noted previously, the *β_CRE_* are FE estimates. The *γ_CRE_* give the difference between the between- and within-person estimates of the time-varying variables *x_it_*. When the cross-time means (*x–_i_*) are removed, the equation becomes

$$y_{it}=\mu_{t}+\beta_{RE}x_{it}+u_{i}+e_{it}.$$

There are two key differences. First, the person-specific error term becomes *u_i_*, which is not allowed to be correlated with the predictors in the model. Second, the coefficients on the time-varying variables *x_it_* are now random-effects estimates, *β_RE_*, which combine within- and between-person variation. As outlined by Wooldridge (2016), the relative contribution of each is given by

(1)
$$\beta_{RE}(x_{it}-\theta {\bar{x}}_{i}).$$

This shows that a portion of the cross-time means is subtracted from their respective time-varying variables, with the portion given by *θ*. *θ* is calculated as

(2)
$$\theta =1-\sqrt{\frac{\sigma_{e}^{2}}{\sigma_{e}^{2}+T\sigma_{u}^{2}}}$$

where *T* is total number of time periods, $\sigma_{e}^{2}$ is the within-person variance, and $\sigma_{u}^{2}$ is the between-person variance.

The key point to take from Equations 1 and 2 is that the RE estimates can be more heavily influenced toward either the within- or between-person estimates depending on whether the *x_it_* have relatively more within- or between-person variation. In cases where there is significantly more between-person variation, estimates will be weighted more heavily toward the between-person estimates.

For example, in Table 1, the FE estimate for HASS majors from the CRE model (Model 1) is .24. The cross-wave mean estimate for HASS majors is –.13, indicating the between-person estimate is .24 – .13 = .11. The RE estimate should be a weighted average of these two. As shown in Model 2, the (rounded) RE estimate is .10, which is about the same as the between-person estimate. The reason the two are so similar is that the HASS variable has much more between- than within-person variation, so the RE estimate is weighted heavily toward the between-person estimate.

The situation is different with the effect of graduate education. Here, the FE estimate is .20, and the between-person estimate is .20 – .22 = −.02. However, the graduate education variable has a fair amount of within-person variation, so the RE estimate does not shift as far toward the between-person estimate and settles at .14.

### Part C: Modeling Selection into Higher Education and Major Field of Study

Previous research on the predictors of college enrollment and major choice informs our selection of controls. Scholarship finds that family socioeconomic status and educational aspirations are important predictors of college enrollment. Owing to greater access to economic and cultural capital, children of higher-income and college-educated parents are more likely to develop the skills needed for admission and to be able to afford the costs (Blau and Duncan 1967; Conley 2001; Jaeger and Breen 2016; Schnittker and Behrman 2012). Parental influence partly operates through value transmission; those who learn to value higher education and become more ambitious in their educational aspirations are more likely to attain higher levels of education (Jaeger and Breen 2016; Vaisey 2010). Research also finds that educational success varies across gender and race, with girls’ cultural capital tending to promote success in schooling, and cultural resources across ethnic groups differentially influencing success (DiMaggio 1982; Dumais 2002; Dumais, Kessinger, and Ghosh 2012; Farkas et al. 1990). Based on this literature, we include several variables related to the socioeconomic status of respondents’ families (parent income, parental education, household size), the skills and cultural capital that promote educational success (respondents’ high school grades, personal educational aspirations, and the importance their parents place on higher education), and sociodemographic differences (gender and race).

Research on major selection suggests both similar and different processes. Major selection is partly an economic decision driven by the anticipated professional outcomes of different degrees (Goyette and Mullen 2006). Family SES thus remains important, with students from more privileged backgrounds being more likely to prioritize non-economic concerns such as personal fulfillment (Astin and Oseguera 2004; Goyette and Mullen 2006). Perceptions of value congruence are also important for major selection (Goyette and Mullen 2006). Value differences sometimes correspond to demographic groups, partly explaining why women and African Americans tend to be underrepresented in STEM but overrepresented in humanities and business administration, respectively (Goyette and Mullen 2006; Hinrichs 2015), and Asians and children of immigrants tend to be overrepresented in STEM but less likely to enroll in business or the social sciences (National Science Foundation 1999; Rangel and Shi 2019). Accordingly, in addition to race and gender, our analysis controls for immigration status. Scholars also differentiate fields according to how they appeal to different personality types (Pike 2006). Degrees in business appeal to students who are more extroverted and less neurotic, whereas the humanities and social sciences appeal to those who are more adventurous and non-conformist (Kaufman, Pumaccahua, and Holt 2013; Pike 2006). Wave 1 of the NSYR did not include personality measurements, but we include controls relevant to extroversion, neuroticism, and conformity—specifically, self-reported popularity among peers, feelings of meaninglessness, and the desire to conform with peers.

Finally, to control for the possibility that college enrollment and major selection is motivated by prior moral attitudes, we include both sociodemographic variables related to moral differences (teen religious affiliation and political ideological background of parents), as well as constructs related to moral concerns. These include religiosity, attitudes toward abstaining from premarital sex, and social concern for marginalized populations. These moral constructs are imperfect as substitutes, but previous literature supports theoretical links between them. Moral concern for order is closely related to religiosity and conservative attitudes (Graham and Haidt 2010; Graham et al. 2009). Social concern for marginalized populations taps into concern for others, with both constructs addressing care for the vulnerable (Forsberg, Nilsson, and Jørgensen 2019; Low and Wui 2016). Although we cannot assess how well these constructs substitute for direct measures of moral concerns at wave 1, we can evaluate their associations at wave 4. We have direct measures of our social concern index, attitudes toward premarital sex, and religiosity—a composite measure including religious beliefs, practices, and the personally felt salience of religion. We use respondent political ideology as a surrogate for parental ideology under the assumption that at wave 1, teen ideology would reflect parental ideology. These variables explain 13 percent of the variation in moral concern for others, and 24 percent of the variation in moral concern for order. On the metric of correlations, these figures correspond to *r* = .36 and *r* = .49, respectively, both of which would be ranked as “moderate” in size under Cohen’s widely used classification scheme (although the correlation for concern for order is borderline large). The higher predictive power of our controls for concern for social order is worth noting given that our analyses indicate higher education only changes this type of moral concern, suggesting we have greater protection from morally motivated selection processes where it is most needed to bolster our results.

Table C1 describes how these variables predict selection into higher education in the NSYR. All these variables were measured at wave 1, and thus prior to enrollment. Model 1 estimates a logistic regression model predicting college enrollment; Models 2 and 3 estimate multinomial logistic regression models predicting the amount of higher education respondents pursue and the majors they select. These models have McFadden *R*^2^ scores of .26, .16, and .07, respectively. Consistent with the literature, nearly every variable included in the model predicts some form of selection at a significant level, thus justifying their inclusion as controls. In fact, the only variables that are not at least marginally significant predictors of selection include several of those included as constructs related to morality, namely moral relativism, attitudes toward premarital sex, and whether a respondent’s parents are liberal or conservative. That these variables are *not* found to be significant predictors of selection, in fact, offers initial evidence that higher education’s effects on subsequent morality are not reducible to selection. Nevertheless, some variables tied to morality do predict enrollment, notably religiosity and social concern for marginalized populations, both of which predict major selection. Therefore, although our results caution against reducing higher education’s effects to selection processes, we cannot rule selection out completely.

**Table C1.** Selection into Higher Education

	**m1:** Logistic Model Predicting Enrollment (baseline: no college)		**m2:** Multinomial Logistic Model Predicting Highest Level of Educational Attainment (baseline: no college)				**m2:** Multinomial Logistic Model Predicting Major Field of Study among Those Enrolled (baseline: STEM)					
	Enrollment		Bachelor’s Degree		Graduate Studies		HASS		Business/Agric.		Education	
Variables	Coef.	SE	Coef.	SE	Coef.	SE	Coef.	SE	Coef.	SE	Coef.	SE
Parent Income	.612***	(.099)	.373***	(.085)	.615***	(.108)	−.101	(.100)	.084	(.109)	−.212	(.195)
Parent Education	.776***	(.176)	−.070	(.222)	.192	(.332)	.396	(.287)	.091	(.325)	.296	(.625)
Household Size	−.157*	(.073)	−.078	(.079)	−.316**	(.110)	.108	(.094)	.183	(.106)	.059	(.163)
Parent Immigrant	1.002**	(.353)	.289	(.261)	.227	(.322)	.129	(.323)	.117	(.339)	−.823	(.619)
Black	.699*	(.343)	.230	(.327)	.144	(.420)	.201	(.444)	.047	(.471)	−1.291*	(.626)
Hispanic	−.210	(.281)	−.832**	(.304)	−.233	(.348)	−.445	(.333)	−.651	(.382)	−.373	(.656)
Asian	1.425	(1.160)	.353	(.686)	1.572*	(.645)	−.206	(.581)	−.430	(.748)	−13.317***	(.622)
Mainline Protestant	.078	(.317)	.368	(.299)	.408	(.395)	−.689	(.356)	−.215	(.412)	.232	(.892)
Catholic	.529	(.286)	.279	(.269)	.324	(.355)	−.722*	(.327)	−.198	(.381)	.381	(.824)
Evangelical Protestant	−.426	(.283)	−.368	(.300)	−.566	(.409)	−1.023**	(.378)	−.804	(.434)	−.184	(.841)
African American Protestant	−.500	(.441)	−.962*	(.462)	−1.066	(.642)	−1.613**	(.614)	−1.133	(.654)	−.060	(1.048)
Jewish	−.662	(.640)	1.261	(.714)	1.377	(.775)	−.001	(.680)	.607	(.733)	.555	(1.366)
Mormon	.115	(.544)	−.518	(.492)	−.855	(.703)	−.867	(.682)	−.498	(.697)	.600	(1.099)
Other Religion	−.603	(.460)	−.408	(.420)	−.155	(.579)	−.601	(.514)	−.854	(.761)	−12.933***	(.893)
Indeterminate Religious Tradition	.352	(.556)	.308	(.564)	.078	(.782)	.754	(1.162)	1.937	(1.192)	−11.934***	(1.294)
Parent Conservative	.164	(.178)	.250	(.167)	.371	(.209)	−.294	(.202)	−.263	(.223)	−.400	(.388)
Parent Liberal	.089	(.207)	.140	(.201)	.209	(.262)	−.092	(.232)	−.339	(.265)	.490	(.454)
Female	.506**	(.156)	−.107	(.147)	.139	(.185)	.418*	(.175)	−.030	(.188)	1.234***	(.353)
Higher Education Important for Parents	.180*	(.072)	.092	(.082)	.232*	(.119)	−.034	(.098)	.118	(.116)	−.125	(.183)
College Aspirations	.671**	(.221)	.717*	(.325)	1.251*	(.602)	−.684	(.487)	−.744	(.538)	−.983	(.879)
Grades in High school	.605***	(.078)	.790***	(.092)	1.216***	(.125)	−.237*	(.107)	−.242*	(.112)	.307	(.214)
Popularity at School	−.075	(.078)	−.105	(.080)	−.082	(.090)	.008	(.088)	.189	(.103)	.229	(.175)
Feelings of Meaninglessness	.031	(.072)	−.149	(.079)	−.190*	(.096)	−.085	(.092)	−.375***	(.102)	.045	(.193)
Conformity	−.138	(.075)	.117	(.077)	−.002	(.092)	−.085	(.092)	.115	(.105)	.067	(.193)
Social Concern	.036	(.078)	.103	(.079)	.027	(.097)	.228*	(.097)	.033	(.098)	−.407*	(.180)
Against Premarital Sex	−.109	(.085)	.014	(.084)	−.119	(.104)	−.081	(.099)	−.066	(.103)	−.049	(.190)
Religiosity	.092	(.103)	.042	(.102)	.254	(.130)	.262*	(.117)	.308*	(.123)	.750***	(.225)
Moral Relativism	−.216	(.153)	.019	(.151)	−.220	(.188)	−.114	(.182)	.030	(.194)	−.007	(.339)
Ever Suspended in High School	−.434*	(.188)	−.776**	(.262)	−.259	(.334)	−.520	(.302)	−.283	(.334)	−1.702	(1.078)
Changed High School	−.493**	(.150)	−.439**	(.149)	−.451*	(.192)	−.215	(.179)	−.107	(.196)	−.387	(.345)
Parent Homeowner	.363*	(.178)	.264	(.214)	.216	(.293)	−.238	(.265)	−.682*	(.283)	.257	(.543)
Constant	.196	(.409)	−.866	(.489)	−2.815***	(.785)	1.825**	(.706)	1.914*	(.774)	−1.766	(1.404)
	*n =* 1,610		*n =* 1,279				*n =* 952					

* *p* < .05; ^**^*p* < .01; ^***^*p* < .001 (two-tailed tests).

### Part D: Full Models

**Table D1.** Higher Education and Moral Foundations

	Moral Foundations									
	Individualizing				Binding					
Variables	Harm		Fairness		Loyalty		Authority		Purity	
*Education Attained (ref. cat.: no educ. beyond HS)*										
Some College	−.053	(.070)	.072	(.071)	−.034	(.069)	−.024	(.062)	−.093	(.062)
Bachelor’s Degree	−.164	(.099)	.003	(.101)	−.144	(.100)	−.059	(.094)	−.134	(.093)
Graduate Studies	−.026	(.110)	.060	(.112)	−.188	(.108)	−.161	(.106)	−.182	(.106)
*Major Field of Study (ref. cat.: no educ. beyond HS)*										
Humanities/Arts/Social Science	.074	(.065)	.054	(.067)	−.174*	(.070)	−.156*	(.071)	−.177*	(.069)
STEM	.008	(.070)	−.035	(.070)	−.050	(.071)	−.054	(.070)	−.071	(.071)
Business/Agriculture	.049	(.076)	−.005	(.077)	.094	(.077)	.115	(.074)	.115	(.075)
Education	.163	(.127)	.152	(.132)	.259	(.134)	.249	(.142)	.323*	(.137)
Parent Income	−.050	(.028)	−.050	(.028)	−.064*	(.028)	−.091**	(.028)	−.058*	(.027)
Parent Education	.159*	(.062)	.101	(.063)	.054	(.064)	−.012	(.058)	−.092	(.055)
Household Size	−.033	(.023)	−.010	(.023)	−.012	(.023)	.015	(.023)	−.004	(.022)
Parent Immigrant	.207*	(.090)	.188*	(.086)	.042	(.090)	.101	(.090)	.114	(.083)
Black	−.120	(.108)	.153	(.108)	−.296**	(.113)	.295**	(.096)	.207*	(.097)
Hispanic	.028	(.089)	.190*	(.091)	−.054	(.091)	.045	(.087)	.097	(.086)
Asian	−.318	(.174)	−.216	(.224)	−.285	(.184)	−.211	(.193)	−.220	(.197)
Mainline Protestant	−.037	(.091)	.014	(.093)	.250**	(.090)	.270**	(.092)	.048	(.091)
Catholic	−.120	(.084)	−.020	(.086)	.193*	(.082)	.167*	(.079)	.084	(.078)
Evangelical Protestant	−.200*	(.090)	−.037	(.092)	.203*	(.092)	.236**	(.084)	.109	(.085)
African American Protestant	−.156	(.141)	.107	(.145)	.091	(.145)	.141	(.127)	.020	(.130)
Jewish	−.049	(.180)	−.089	(.162)	.064	(.170)	.203	(.152)	.036	(.155)
Mormon	−.051	(.155)	−.026	(.153)	.312*	(.142)	.440**	(.139)	.353*	(.149)
Other Religion	−.061	(.147)	.082	(.149)	−.372*	(.148)	−.022	(.153)	.076	(.165)
Indeterminate Religious Tradition	.017	(.163)	.229	(.165)	.353*	(.162)	.370*	(.144)	−.003	(.153)
Parent Conservative	−.086	(.052)	−.089	(.052)	−.011	(.052)	.056	(.049)	.071	(.048)
Parent Liberal	.048	(.060)	.199**	(.061)	−.073	(.060)	−.052	(.061)	−.043	(.061)
Female	.269***	(.046)	.066	(.047)	−.101*	(.046)	.122**	(.043)	.217***	(.044)
Higher Education Important for Parents	.023	(.024)	.024	(.024)	.017	(.022)	−.014	(.022)	.021	(.022)
College Aspirations	.022	(.080)	−.036	(.081)	−.069	(.077)	−.037	(.076)	−.158*	(.071)
Grades in High School	−.024	(.028)	−.023	(.029)	−.039	(.028)	−.021	(.026)	−.049	(.026)
Popularity at School	−.000	(.024)	.020	(.024)	.092***	(.024)	.094***	(.023)	.126***	(.023)
Feelings of Meaninglessness	−.028	(.023)	−.028	(.022)	−.059*	(.023)	−.027	(.022)	−.049*	(.021)
Conformity	−.017	(.023)	.002	(.024)	.057*	(.023)	.081***	(.022)	.043*	(.022)
Social Concern	.118***	(.024)	.103***	(.024)	−.011	(.022)	−.023	(.022)	−.013	(.022)
Against Premarital Sex	.069**	(.025)	.043	(.026)	.049	(.026)	.054*	(.025)	.080**	(.025)
Religiosity	.018	(.031)	−.011	(.031)	.060*	(.031)	.159***	(.030)	.175***	(.029)
Moral Relativism	.044	(.046)	.010	(.046)	.021	(.045)	−.036	(.045)	.012	(.044)
Ever Suspended in High School	−.118	(.066)	−.170**	(.066)	−.007	(.064)	−.082	(.061)	−.021	(.058)
Changed High School	.042	(.046)	.036	(.046)	−.048	(.044)	−.017	(.044)	.053	(.044)
Parent Homeowner	.135*	(.064)	.076	(.064)	.108	(.063)	.073	(.060)	.121*	(.059)
Constant	−.242	(.138)	−.232	(.140)	.004	(.137)	−.214	(.126)	−.028	(.124)

*Note: N* = 2,012.

* *p* < .05; ^**^*p* < .01; ^**^*p* < .001 (two-tailed tests).

**Table D2.** Linear Regressions of Moral Concern on Educational Attainment and Fields of Study

	Moral Concern			
Variables	For Others		For Social Order	
*Education Attained (ref. cat.: no educ. beyond HS)*				
Some College	.009	(.071)	−.067	(.062)
Bachelor’s Degree	−.093	(.101)	−.140	(.094)
Graduate Studies	.019	(.111)	−.215*	(.105)
*Major Field of Study (ref. cat.: no educ. beyond HS)*				
Humanities/Arts/Social Science	.072	(.065)	−.203**	(.071)
STEM	−.016	(.068)	−.073	(.072)
Business/Agriculture	.026	(.075)	.132	(.076)
Education	.177	(.124)	.341*	(.136)
Parent Income	−.056*	(.027)	−.085**	(.027)
Parent Education	.147*	(.063)	−.025	(.058)
Household Size	−.025	(.023)	−.002	(.022)
Parent Immigrant	.225*	(.090)	.106	(.087)
Black	.013	(.107)	.096	(.097)
Hispanic	.118	(.091)	.038	(.087)
Asian	−.305	(.187)	−.292	(.176)
Mainline Protestant	−.011	(.091)	.216*	(.089)
Catholic	−.081	(.084)	.177*	(.077)
Evangelical Protestant	−.135	(.089)	.214*	(.085)
African American Protestant	−.038	(.141)	.100	(.128)
Jewish	−.075	(.171)	.116	(.158)
Mormon	−.041	(.151)	.444**	(.144)
Other Religion	.008	(.145)	−.115	(.148)
Indeterminate Religious Tradition	.134	(.160)	.274	(.149)
Parent Conservative	−.100	(.052)	.049	(.049)
Parent Liberal	.132*	(.059)	−.068	(.059)
Female	.193***	(.047)	.106*	(.043)
Higher Education Important for Parents	.026	(.024)	.010	(.022)
College Aspirations	−.004	(.083)	−.112	(.072)
Grades in High school	−.026	(.029)	−.045	(.026)
Popularity at School	.010	(.024)	.128***	(.023)
Feelings of Meaninglessness	−.031	(.023)	−.055*	(.021)
Conformity	−.009	(.024)	.073***	(.022)
Social Concern	.126***	(.024)	−.019	(.022)
Against Premarital Sex	.064*	(.025)	.075**	(.025)
Religiosity	.004	(.031)	.166***	(.029)
Moral Relativism	.032	(.046)	.001	(.044)
Ever Suspended in High School	−.164*	(.066)	−.045	(.060)
Changed High School	.043	(.046)	.000	(.043)
Parent Homeowner	.120	(.064)	.126*	(.060)
Constant	−.268	(.138)	−.094	(.126)

*Note: N* = 2,012.

* *p* < .05; ^**^*p* < .01; ^***^*p* < .001 (two-tailed tests).

**Table D3.** Moral Change for HASS Majors across Parental Ideology

	Moral Relativism		Moral Progressivism		Moral Concern for Social Order	
Variables	Coef.	SE	Coef.	SE	Coef.	SE
*Education*						
Degree-Holder	−.120	(.061)	.073	(.060)	−.183	(.145)
HASS	−.124*	(.058)	.093	(.061)	−.395**	(.132)
STEM	−.115**	(.043)	−.037	(.046)	−.092	(.114)
Business/Agriculture	−.072	(.042)	.050	(.047)	.122	(.121)
Education	−.118	(.083)	.018	(.092)	.310*	(.151)
Parent Liberal	.095*	(.047)	.072	(.051)	−.161	(.111)
Parent Conservative	−.149***	(.039)	−.200***	(.042)	−.112	(.111)
*Interaction Effects*						
Degree-Holder × Liberal Parents	.047	(.094)	.011	(.091)	−.126	(.162)
Degree-Holder × Conservative Parents	.058	(.073)	−.070	(.071)	.070	(.140)
HASS × Liberal Parents	.103	(.101)	.088	(.103)	.342*	(.173)
HASS × Conservative Parents	−.036	(.080)	−.036	(.089)	.314*	(.138)
Constant	.233***	(.068)	.097	(.067)	−.054	(.156)

*Note*: Moral relativism sample: *N* = 2,180, Obs. = 6,540; moral concern for social order sample: *N* = 1,313. Table only displays main variables, although same controls were used as previous models.

* *p* < .05; ^**^*p* < .01; ^***^*p* < .001 (two-tailed tests).

## Notes

1. Although the particulars of how SES and morality relate are not always consistent across studies, SES is generally positively tied to a liberal moral profile characterized by a focus on individuals and openness to new experience, and negatively associated with traditional forms of morality such as group loyalty and valuing authority.

2. Moral relativism is available at wave 1; however, it was measured as a binary variable rather than using a four-point scale as in later waves. Therefore, we restrict our analyses of moral relativism to waves 2, 3, and 4.

3. We omitted cases where the highest level of education was an associate’s degree/vocational/technical certificate, given the low number of cases (*n* = 52) and the lack of theoretical expectations for this group.

4. Survey weights must be included if a sampling design is informative—that is, the composition of the sample contains information about the relationship between the independent and dependent variables. If the sampling design in non-informative, including survey weights will not change the coefficients much, but might increase the standard errors (Solon et al. 2015). Our results were similar with and without weights and invariably gave the same substantive conclusions, suggesting the sampling design is not informative for our research questions. As a further test, we examined whether the survey weights predicted our moral outcome variables, net of other controls. In all cases, sampling weights were non-significant, indicating they did not add information about the outcomes to our analyses. Consequently, we did not use survey weights in our analyses. This increased the efficiency of our estimates and allowed us to use cases that were missing sampling weights at a given wave, thus increasing our final sample size.

5. The low levels of moral progressivism among education majors appear to contradict the socialization hypothesis, in that education is often depicted as a highly liberal field. However, the sample of education majors is quite small (*n* = 58), so it is possible our sample does not reflect general trends in this field.

6. In some cases, effects for educational variables changed a great deal between the CRE and non-FE models. This is because the non-FE models calculate effects using both within- and between-person variation. To the extent that between-person effects differ from within-person effects, the resulting coefficient is a weighted average between the two, with the weights corresponding to the relative amounts of within- and between-person variation for the variable in question. Several education variables had small amounts of within-person variation, which made them more susceptible to change. See Appendix B for a further discussion of this issue.

7. These results remain robust even when a fuller set of controls is included in the models. These are the same controls discussed in Appendix C and used in models for predicting moral concern for others and for social order.

8. For instance, a teen who identifies with a religious denomination and attends church at least once a week is, on average, .96 SD higher on religiosity at wave 1 than a teen who is non-religious and does not attend church weekly.

9. It is possible that although overall endorsement of concern for others does not change, how this concern is understood and applied does. For example, concern for friends and family might be broadened to include concern for strangers, particular social groups (e.g., marginalized groups), or the environment. Similarly, moral concern for others might be linked to an expanded repertoire of behaviors, such as monitoring oneself and others for signs of harmful beliefs and behaviors, or various forms of political activism. Our data do not allow us to assess whether these types of changes are occurring.

10. Examining effects by ideological subgroups has two other advantages. First, it allows us to determine whether the moral changes associated with HASS degrees co-occur in the same students. This is important for isolating the processes underlying the effects. For instance, if decreased concern for social order coincides with increased moral absolutism and moral progressivism, this is consistent with a moral socialization hypothesis. If, however, students from conservative homes show an increase in moral absolutism without decreasing concern for social order, it might suggest a hardening of prior moral commitments in the face of contradictory moral influences. Subgroup analysis also provides a more rigorous control for selection processes. Parents’ ideologies likely affect the universities their children choose to attend, which, in turn, could influence the experiences students have in particular majors. For instance, it seems plausible that course readings and discussions about race, sexuality, and other sensitive topics would differ between liberal and conservative universities.

11. For example, degrees in business/agriculture or education majors can increase moral certitude influencing moral concern for social order in a liberal direction.

12. Emerging initiatives advanced by college administrators related to trigger warnings, microaggressions, and safe spaces, for example, appear to exemplify liberal harm-reduction as the cardinal moral criteria (Campbell and Manning 2018; Lukianoff and Haidt 2018). In professional workplaces, moreover, Dobbin and Kalev (2013) carefully document the diffusion of diversity initiatives advanced by corporate personnel experts in large firms since at least the 1980s. Some question the sincerity of these initiatives, arguing they are often symbolic gestures that leave structural inequalities intact (Berrey 2015; Collins 2011). Be that as it may, putative changes in professional culture dovetail trends among employees in higher education and professional sectors toward the political left more generally (Gelman 2008; OpenSecrets.org 2021; Piketty 2020; see also Gu 2020). As such, institutional endorsement of social justice may combine with selection processes of “cultural fit” (Rivera 2012) to contribute to a professional culture that is increasingly liberal in its sensibilities.

13. This might partly explain Trump’s lower support among the college-educated. Not only did Trump flout cosmopolitan moral norms, but he also exhibited an ambivalent attitude toward what many people considered to be objective truth. Repeated allegations of “fake news” and the use of “alternative facts” are unlikely to have endeared him to a professional class that believes definite truths do exist, especially if education and expertise are seen as critical to discerning and applying those truths.

14. At first glance, this account of morally-motivated conflict appears at odds with recent work by Baldassarri and Park (2020), who argue that moral attitudes are not polarizing because both Democrats and Republicans adopt more liberal moral attitudes over time but at different paces. We see our results as complementing their work by suggesting that colleges and universities might play an important role in the early diffusion of moral ideas. Higher education can encourage liberal moral attitudes and facilitate the formation of networks of like-minded peers through which new moral views can spread (Dey 1996, 1997; Strother et al. 2020). As graduates from these programs become professional elites, their ideas may “trickle down,” becoming a form of cultural capital that spreads as it is emulated by others (Bourdieu 1984; Campbell and Manning 2018; Simmel 1957). In this way, higher education can facilitate the convergence of moral attitudes in the long-term, even while provoking tension in the short-term as a vanguard for moral change.
